# Aspartate signaling drives lung metastasis via alternative translation

**DOI:** 10.1038/s41586-024-08335-7

**Published:** 2025-01-01

**Authors:** Ginevra Doglioni, Juan Fernández-García, Sebastian Igelmann, Patricia Altea-Manzano, Arnaud Blomme, Rita La Rovere, Xiao-Zheng Liu, Yawen Liu, Tine Tricot, Max Nobis, Ning An, Marine Leclercq, Sarah El Kharraz, Panagiotis Karras, Yu-Heng Hsieh, Fiorella A. Solari, Luiza Martins Nascentes Melo, Gabrielle Allies, Annalisa Scopelliti, Matteo Rossi, Ines Vermeire, Dorien Broekaert, Ana Margarida Ferreira Campos, Patrick Neven, Marion Maetens, Karen Van Baelen, H. Furkan Alkan, Mélanie Planque, Giuseppe Floris, Albert Sickmann, Alpaslan Tasdogan, Jean-Christophe Marine, Colinda L.G.J. Scheele, Christine Desmedt, Geert Bultynck, Pierre Close, Sarah-Maria Fendt

**Affiliations:** 1 Laboratory of Cellular Metabolism and Metabolic Regulation, VIB Center for Cancer Biology, VIB, Herestraat 49, 3000 Leuven, Belgium; 2 Laboratory of Cellular Metabolism and Metabolic Regulation, Department of Oncology, KU Leuven and Leuven Cancer Institute (LKI), Herestraat 49, 3000 Leuven, Belgium; 3 Laboratory of Cancer Signaling, GIGA-Institute, University of Liège, Liège, Belgium; 4 Laboratory of Molecular & Cellular Signaling, Department of Cellular & Molecular Medicine, KU Leuven, Herestraat 49, 3000; 5 Department of Gastroenterology, Affiliated Hospital of Jiangsu University, Jiangsu University, Zhenjiang, China; 6 Laboratory of Intravital Microscopy and Dynamics of Tumor Progression, VIB Center for Cancer Biology, VIB, Herestraat 49, 3000 Leuven, Belgium; 7 Laboratory of Intravital Microscopy and Dynamics of Tumor Progression, Department of Oncology, KU Leuven, Herestraat 49, 3000 Leuven, Belgium; 8 Intravital Imaging Expertise Center, VIB Center for Cancer Biology, VIB, Herestraat 49, 3000 Leuven, Belgium; 9 Laboratory for Molecular Cancer Biology, VIB Center for Cancer Biology, VIB, Herestraat 49, 3000 Leuven, Belgium; 10 Laboratory for Molecular Cancer Biology, Department of Oncology, KU Leuven, Herestraat 49, 3000 Leuven, Belgium; 11 Leibniz Institut für Analytische Wissenschaften-ISAS-e.V., Dortmund, Germany; 12 Department of Dermatology, University Hospital Essen and German Cancer Consortium, Essen, Germany; 13 Department of Gynaecology and Obstetrics, UZ Leuven, Herestraat 49, 3000 Leuven, Belgium; 14 Laboratory for Translational Breast Cancer Research, Department of Oncology, KU Leuven, Herestraat 49, 3000 Leuven, Belgium; 15 Spatial Metabolomics Expertise Center, VIB Center for Cancer Biology, VIB, Herestraat 49, 3000 Leuven, Belgium; 16 Department of Pathology, UZ Leuven, Herestraat 49, 3000 Leuven, Belgium; 17 Department of Imaging and Pathology, Laboratory of translational cell and tissue research, KU Leuven, Herestraat 49, 3000 Leuven, Belgium; 18 Department of Chemistry, College of Physical Sciences, University of Aberdeen, Aberdeen, Scotland, United Kingdom; 19 Medizinische Fakultät, Medizinische Proteom-Center (MPC), Ruhr-Universität Bochum, Bochum, Germany; 20 WELBIO department, WEL Research Institute, Avenue Pasteur 6, 1300 Wavre, Belgium

## Abstract

Lung metastases occur in up to 54% of patients with metastatic tumors^1,2^. Contributing factors to this high frequency include the physical properties of the pulmonary system and a less oxidative environment that may favor the survival of cancer cells^3^. Moreover, secreted factors from primary tumors alter immune cells and the extracellular matrix of the lung creating a permissive pre-metastatic environment primed for the arriving cancer cells^4,5^. Recently, it was shown that also nutrients are primed during pre-metastatic niche formation^6^. Yet, whether and how nutrients available in future organs of metastases confer cancer cells with aggressive traits is mostly undefined. Here, we discovered that pulmonary aspartate triggers a cellular signaling cascade in disseminated cancer cells resulting in a translational program that boosts lung metastasis aggressiveness. Specifically, we observe that patients and mice with breast cancer have high concentrations of aspartate in their lung interstitial fluid. This extracellular aspartate activates the ionotropic N-methyl-D-aspartate (NMDA) receptor in cancer cells, which induces CREB-dependent expression of deoxyhypusine hydroxylase (*DOHH*). The latter is essential for hypusination, a posttranslational modification required for the activity of the non-classical translation initiation factor eIF5A. In turn, a translational program with TGF-β signaling as a central hub promotes collagen synthesis in lung-disseminated breast cancer cells. We detect key proteins of this mechanism in lung metastases from patients with breast cancer. In summary, we discover that aspartate, a classical biosynthesis metabolite, functions in the lung environment as an extracellular signaling molecule promoting metastasis aggressiveness.

## Alternative translation promotes aggressiveness

**Extended Data Figure 1:**
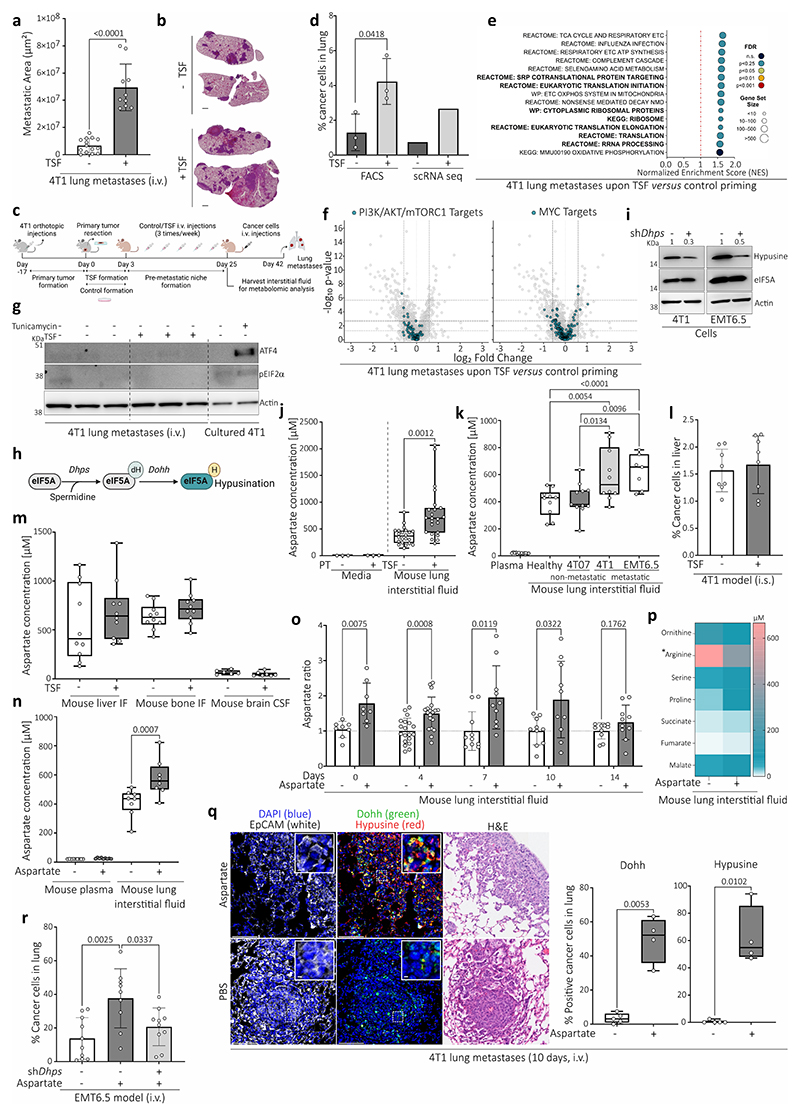
Aggressive lung metastases display increased translation independent of classical regulators or stress stimuli. **a**. Total lung metastatic area 17 days after i.v. injections with 4T1 cells in mice pre-treated with tumor secreted factors (TSF, *n* = 10) or control medium (*n* = 14). Data are presented as mean ± sd. Unpaired two-tailed *t*-test with Welch correction. Representative images are depicted in **b**. **b**. Representative H&E stainings from **a**., two individual lung lobes from one mouse each were selected with automatic tissue detection algorithm in Zen Software and pasted in a white background, scale bars = 1 mm. **c**. Schematic representation of the pre-metastatic niche formation and lung metastasis model. TSF, Tumor secreted factors; i.v., intravenous injection. **d**. Percentage of cancer cells present in the lung 16 days after i.v. injection with CD90.1^+^ 4T1 cells in mice pre-treated with TSF or control medium, measured by flow cytometry (FACS, *n* = 3 mice) and scRNA-seq (pool of *n* = 3 mice). FACS data are presented as mean ± s.d. Unpaired two-tailed *t*-test with Welch correction. **e**. GSEA normalized enrichment scores (NES) for the top 15 upregulated gene sets found on cancer cells based on scRNA-seq comparing 4T1 lung metastases from mice pre-treated with control medium or TSF. Dot colors and areas indicate FDR-adjusted *P-*values and gene-set sizes, respectively. Gene sets related to translation are highlighted in bold. *P-*values based on *fgsea*’s adaptive multilevel splitting Monte Carlo approach, subject to FDR adjustment using the Benjamini-Hochberg (BH) approach. **f**. Volcano plots based on single-cell differential expression analysis comparing cancer cells in 4T1 lung metastases from mice pre-treated with control medium or TSF. All genes in the Hallmark PI3K-AKT-MTOR signaling gene set (left) and either of the Hallmark MYC targets (V1/V2) gene sets (right) are highlighted in blue. Log_2_ Fold Changes (FC) and negative log_10_-transformed *P*-values are indicated in the x and y axes respectively. The horizontal dashed lines represent (from bottom to top) raw, BH-adjusted and Bonferroni adjusted *P*-values of 0.05, while the vertical ones represent absolute fold-changes of 1.5. *P-*values based on Seurat’s Wilcoxon rank-sum test implementation. **g**. ATF4 and phosphorylated eIF2α levels in 4T1 lung metastases in mice pre-treated with TSF (*n* = 3) or control medium (*n* = 3), and in cultured 4T1 cells treated with or without tunicamycin induced ER-stress. **h**. Schematic representation of eIF5A hypusination pathway. Enzymes are indicated in italics. Solid lines represent single reactions. dH = deoxyhypusine; H = hypusine. **i**. Hypusine levels in 4T1 (left) or EMT6.5 (right) cells silenced for *Dhps* or scramble shRNA. Quantification of hypusine signal normalized over total eIF5A signal is indicated on top of each lane. **j**. Box and whisker plots of aspartate concentrations in control- or primary tumor (PT)-conditioned medium and lung interstitial fluid of mice pre-treated with TSF (*n* = 23) or control medium (*n* = 22). Box hinges indicate the 1^st^/3^rd^ quartiles of the corresponding data, while box mid-lines represent medians, and whiskers span the range of the data. Individual data points are indicated by the white dots. One-way ANOVA (*P* < 0.0001) with Tukey’s multiple-comparison tests. **k**. Box and whisker plots of aspartate concentrations in the blood plasma and lung interstitial fluid of healthy mice or mice injected (m.f.p.) with 4T07, 4T1 or EMT6.5 breast cancer cells, 17 days after injection (plasma, *n* = 13; healthy, *n* = 10; 4T07, *n* = 11; 4T1, *n* = 10; EMT6.5, *n* = 7). Box hinges indicate the 1^st^/3^rd^ quartiles of the corresponding data, while box mid-lines represent medians, and whiskers span the range of the data. Individual data points are indicated by the white dots. One-way ANOVA (*P* < 0.0001) with Tukey’s multiple-comparison tests; *P* < 0.0001 between plasma and all lung interstitial fluid conditions. **l**. Percentage of cancer cells present in the liver 14 days after intrasplenic (i.s.) injections with CD90.1^+^ 4T1 cells in mice pre-treated with TSF (*n* = 8) or control medium (*n* = 8). Data are presented as mean ± sd. Unpaired two-tailed *t*-test with Welch correction. I.s. injection of 4T1 cancer cells did not yield a change in liver metastases in TSF-treated mice compared to control mice. **m**. Box and whisker plots of aspartate concentrations in the liver and bone interstitial fluid and brain cerebrospinal fluid of mice pre-treated with TSF or control medium (liver, *n* =10 control, *n* = 10 TSF; bone, *n* = 10 control, *n* = 10 TSF; brain, *n* = 6 control, *n* = 7 TSF). Box hinges indicate the 1^st^/3^rd^ quartiles of the corresponding data, while box mid-lines represent medians, and whiskers span the range of the data. Individual data points are indicated by the white dots. Two-way ANOVA with Šídák’s multiple-comparison tests. No statistically significant changes are observed upon TSF treatment for any of the organs. **n**. Box and whisker plots of aspartate concentrations in the blood plasma and lung interstitial fluid of mice pre-treated with daily injections of aspartate (20 mM, i.p., 10 days, *n* = 8) or PBS (*n* = 8). Box hinges indicate the 1^st^/3^rd^ quartiles of the corresponding data, while box mid-lines represent medians, and whiskers span the range of the data. Individual data points are indicated by the white dots. Two-way ANOVA with Tukey’s multiple-comparison tests; *P* < 0.0001 between all plasma and all lung interstitial fluid conditions, no statistically significant changes are observed upon aspartate treatment in blood plasma. **o**. Ratio of aspartate concentrations in the lung interstitial fluid of mice pre-treated with daily injections of aspartate (20 mM, i.p., 10 days) *vs* PBS. Days indicate the time of interstitial fluid collection after the last injection of aspartate or PBS. Data are presented as mean ± sd. Unpaired two-tailed *t*-test with Welch correction at each time point. **p**. Average metabolite concentrations in the lung interstitial fluid of mice pre-treated with daily injections of aspartate (20 mM, i.p., 10 days, *n* ≥ 8) or PBS (*n* ≥ 8). * indicates *P* = 0.00174. All other metabolites show no statistically significant changes. Unpaired two-tailed *t*-test with Welch correction between PBS-treated and aspartate-treated mice. **q**. Hypusine and Dohh detected in lung metastases 10 days after i.v. injections with 4T1 cancer cells, in mice pre-treated with daily injections of aspartate (20 mM, i.p., 10 days) or PBS, assessed by multiplex immunohistochemistry. Left: representative images from *n* = 5 4T1 PBS, *n* = 4 4T1 aspartate independent mice are shown. White = EpCAM; red = Hypusine; green = Dohh; blue = DAPI nuclear staining. Scale bars = 100 μm. Scale bars zoom-in = 5 μm. The corresponding H&E staining is represented on the right of the panel. Right: quantification of lung metastasis cells positive for Dohh or Hypusine. Box hinges indicate the 1^st^/3^rd^ quartiles of the corresponding data, while box mid-lines represent medians, and whiskers span the range of the data. Individual data points are indicated by the white dots. Unpaired two-tailed *t*-test with Welch correction. **r**. Percentage of cancer cells present in the lung 14 days after i.v. injections with CD90.1^+^ EMT6.5 cells silenced for *Dhps* or scramble shRNA in mice pre-treated with daily injections of aspartate (20 mM, i.p., 10 days) or PBS. *N* = 10 shSCR PBS, *n* = 9 shSCR aspartate, *n* = 10 sh*Dhps* aspartate. Data are presented as mean ± sd. One-way ANOVA (*P* = 0.0029) with Tukey’s multiple-comparison tests.

**Figure 1:**
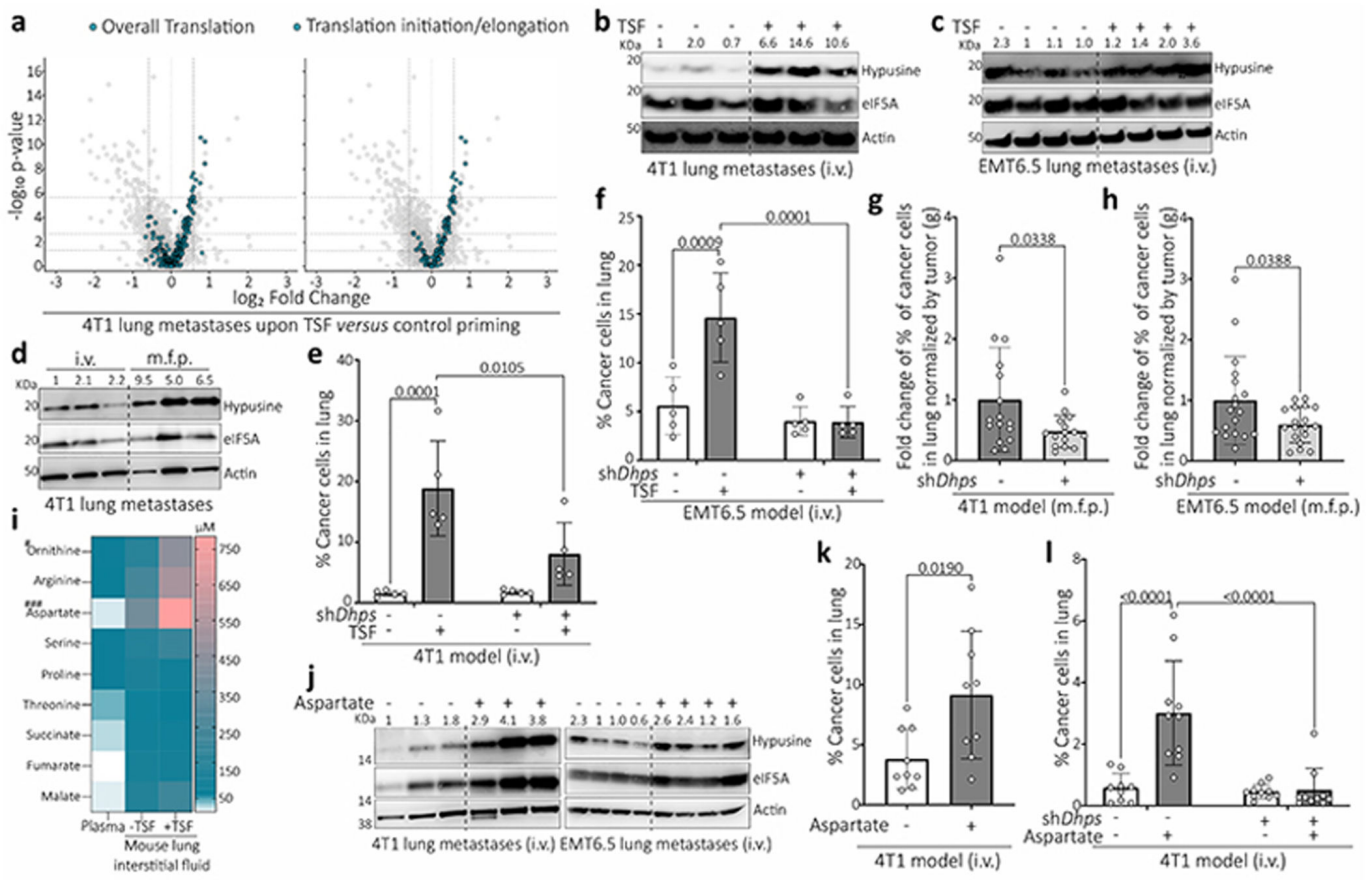
Aspartate promotes lung metastasis aggressiveness by inducing eIF5A hypusination **a**. Volcano plots based on single-cell differential expression analysis comparing cancer cells from control-/TSF-pre-treated 4T1 lung metastases. Genes related to translation (left) and translation elongation/initiation (right) highlighted in blue. Horizontal lines (bottom to top): raw, BH-adjusted and Bonferroni adjusted *P*-values of 0.05. Vertical lines: absolute fold-changes of 1.5. **b,c**. Hypusine levels in 4T1 (**b**) or EMT6.5 (**c**) metastases in mice pre-treated with TSF or control (*n*=3/4 per group for 4T1/EMT6.5, respectively). **d**. Hypusine levels in 4T1 metastases (i.v., *n*= 3, m.f.p., *n*=3). **e,f**. Percentage of CD90.1^+^ 4T1 (**e**) or EMT6.5 (**f**) cells silenced for *Dhps* or scramble shRNA (*n*=5 per group) in mice pre-treated with TSF or control. **g,h**. Fold-change of the percentage of CD90.1^+^ 4T1 (**g**) or EMT6.5 (**h**) cells silenced for *Dhps* (*n*=15, 4T1; *n*=18, EMT6.5) or scramble shRNA (*n*=16, 4T1; *n*=18 EMT6.5) normalized by PT weight (g). Data from 3 (4T1) or 2 (EMT6.5) independent experiments, and normalized relative to each scramble-group average. **i**. Average metabolite concentrations in plasma and lung interstitial fluid in mice pre-treated with TSF or control (*n*≥5 per group). ### *P*=0.0006, # *P*=0.053. **j**. Hypusine levels in 4T1 (left, *n*=3 per group) or EMT6.5 (right, *n*=4 per group) metastases in mice pre-treated with aspartate or PBS. **k**. Percentage of CD90.1^+^ 4T1 cells expressing scramble shRNA (*n*=9 per group) in mice pre-treated with aspartate or PBS. **l**. Percentage of CD90.1^+^ 4T1 cells silenced for *Dhps* or scramble shRNA (*n*=9 shSCR PBS,*n*=10 for all other groups) in mice pre-treated with aspartate or PBS. For **a**, *P* values based on Seurat’s Wilcoxon rank-sum test implementation. For **b,c,d,j**, Hypusine quantification relative to total eIF5A shown on top of each lane. For **e,f,l**, *P*-values based on Two-way ANOVA with Tukey’s multiple-comparison tests. For **g,h,i,k**, *P*-values based on unpaired two-tailed *t*-test with Welch correction. Data presented as mean±sd unless noted.

To investigate lung metastasis aggressiveness, we performed single-cell RNA-sequencing (scRNA-seq) of metastases growing in healthy lungs and in lungs primed with tumor-secreted factors (TSF) from 4T1 breast tumors, which results in a more aggressive metastatic disease (Extended Data Figure 1a-d). Focusing on the cancer cell populations we discovered that several gene sets indicative of increased translation were enriched in the metastases from TSF treated mice (Figure 1a, Extended Data Figure 1e). After excluding an involvement of classical regulators of translation (Extended Data Figure 1f, g), we investigated non-classical activators of translation. We discovered an increased activity of the translation initiation/elongation factor eIF5A^7^ based on its hypusination -an activating posttranslational modification exclusively present on eIF5A^8^ (Extended Data Figure 1h)- in aggressive 4T1 and EMT6.5 lung metastases from TSF treated mice and in spontaneous 4T1 metastases compared to control (Figure 1b-d). To investigate whether eIF5A activity drives metastasis aggressiveness, we reduced eIF5A hypusination by targeting its first enzymatic step catalyzed by deoxyhypusine synthase (DHPS, Extended Data Figure 1h,i). Indeed, silencing *Dhps* in 4T1 and EMT6.5 breast cancer cells abrogated the metastatic aggressiveness induced by TSF and impaired spontaneous metastasis growth (Figure 1e-h). Thus, we concluded that lung metastasis aggressiveness is mediated by eIF5A hypusination.

## Aspartate induces eIF5A hypusination

To identify the trigger of eIF5A hypusination in aggressive metastases, we analyzed the nutrient concentrations in lung interstitial fluid from TSF-primed versus control mice. Most nutrient concentrations did not change significantly (Figure 1i). An exception was aspartate, an amino acid that is very low in blood plasma and that is mostly *de novo* synthesized in cancer cells for the biosynthesis of nucleotides and proteins. In TSF-primed mice or in the presence of a primary breast tumor, lung aspartate increased to almost millimolar levels (Figure 1i, Extended Data Figure 1j,k). This increase did not occur in other organs or in mice with non-metastatic primary breast tumors (Extended Data Figure 1k-m). Next, we investigated whether aspartate induces eIF5A hypusination and lung metastasis aggressiveness. We pre-treated mice with aspartate or PBS (vehicle; i.p.) for 10 days, which resulted in a similar increase in aspartate in lung interstitial fluid as TSF treatment, without changing circulating aspartate (Extended Data Figure 1n-p). Subsequently, we injected the mice with 4T1 or EMT6.5 cancer cells. Metastases from aspartate pre-treated mice displayed elevated eIF5A hypusination (Figure 1j, Extended Data Figure 1q) and increased metastasis aggressiveness based on the total number of cancer cells detected in the lung (Figures 1k). Moreover, decreasing eIF5A hypusination with *Dhps* silencing abrogated aspartate-induced metastasis aggressiveness (Figure 1l, Extended Data Figure 1r). Thus, we concluded that lung aspartate triggers eIF5A hypusination and promotes metastasis aggressiveness.

## Lung-like medium promotes aggressiveness

**Extended Data Figure 2:**
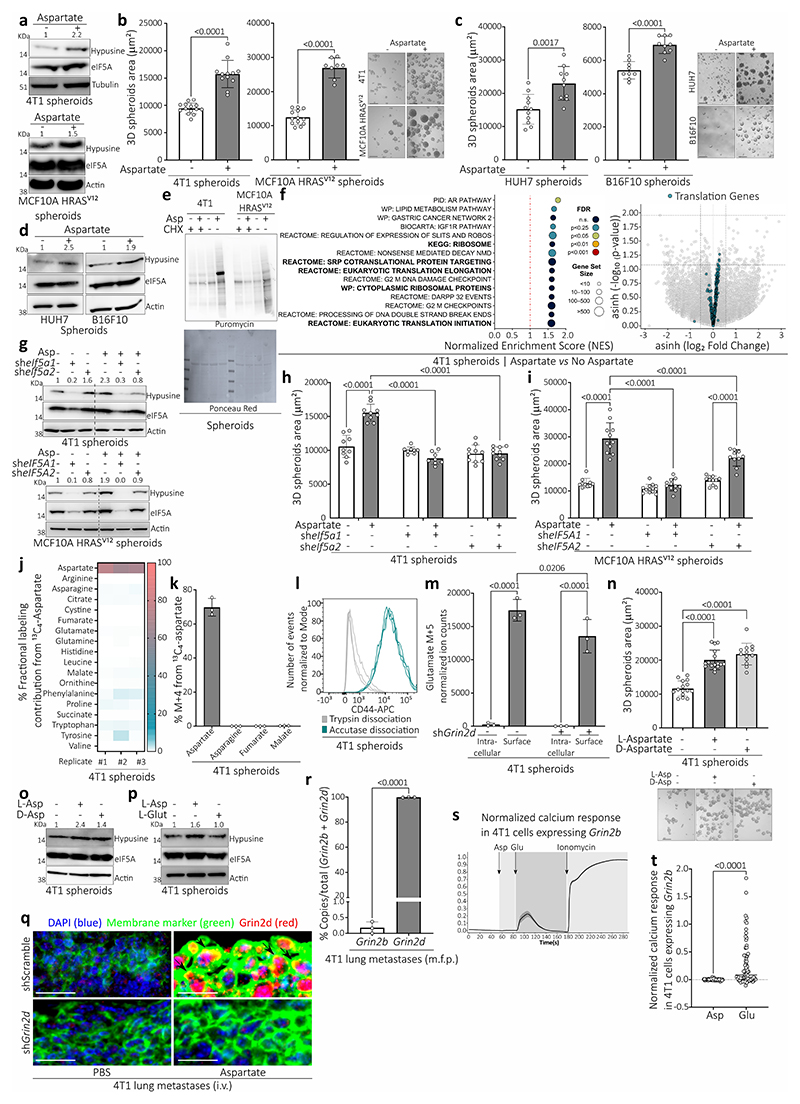
Pulmonary aspartate increases lung metastasis aggressiveness via NMDA receptor activity **a**. Hypusine levels in 4T1 (top) and MCF10A H-Ras^v12^ (bottom) spheroids grown in lung-like medium (LLM) supplemented with or without aspartate. A representative image of *n* = 3 independent experiments is shown. Quantification of hypusine signal normalized over total eIF5A signal is indicated on top of each lane. **b**. Total spheroid areas for 4T1 (left) and MCF10A H-Ras^v12^ (right) cells grown in LLM supplemented with or without aspartate (*n* = 14 4T1 no aspartate, *n* = 13 MCF10A H-Ras^v12^ no aspartate, *n* = 12 4T1 aspartate, *n* = 8 MCF10A H-Ras^v12^ aspartate). Data are presented as mean ± s.d. (*n* indicates independent samples). Unpaired two-tailed *t*-test with Welch correction. Representative images are shown on the right. Scale bar = 250 μm. **c**. Total spheroid areas for HUH7 (left) and B16F10 (right) cells grown in LLM supplemented with or without aspartate. *N* = 11 HUH7 no aspartate, *n* = 9 B16F10 no aspartate, *n* = 10 HUH7 aspartate, *n* = 9 B16F10 aspartate. Data are presented as mean ± s.d. (*n* indicates independent samples). Unpaired two-tailed *t*-test with Welch correction. Representative images are shown on the right. Scale bar = 250 μm. **d**. Hypusine levels in HUH7 (left) and B16F10 (right) spheroids grown in LLM supplemented with or without aspartate. A representative image of *n* = 3 independent experiments is shown. Quantification of hypusine signal normalized over total eIF5A signal is indicated on top of each lane. **e**. Top: SUnSET assay showing puromycin incorporation in 4T1 and MCF10A H-Ras^v12^ spheroids grown in LLM supplemented with or without aspartate. Spheroids were treated with cycloheximide (CHX, 100 nM) or vehicle, followed by puromycin (10 μg/ml). Bottom: Ponceau red staining showing equal protein loading. **f**. Left: GSEA normalized enrichment scores (NES) for the top 15 upregulated gene sets based on total RNA sequencing data for 4T1 spheroids grown in LLM supplemented with aspartate *vs* no aspartate. Dot colors and areas indicate FDR-adjusted *P*-values and gene-set sizes, respectively. Gene sets related to translation are highlighted in bold. Right: Volcano plot based on differential expression analysis of total RNA sequencing data for 4T1 spheroids grown in LLM supplemented with or without aspartate. Genes in Reactome’s translation gene set are highlighted in blue. Log_2_ Fold Changes (FC) and negative log_10_-transformed *P*-values are indicated in the x and y axes respectively. Both axes were further subject to inverse hyperbolic sine transformation for improved visualization. The horizontal dashed lines represent (from bottom to top) raw and BH-adjusted *P*-values of 0.05, while the vertical ones represent absolute fold-changes of 1.5. *P-*values based on *fgsea*’s adaptive multilevel splitting Monte Carlo approach, subject to FDR adjustment using the Benjamini-Hochberg (BH) approach. **g**. Hypusine levels in 4T1 (top) and MCF10A H-Ras^v12^ (bottom) spheroids silenced for *eIf5a1, eIf5a2*, or scramble shRNA, grown in LLM supplemented with or without aspartate. A representative image of *n* = 3 independent experiments is shown. Quantification of hypusine signal normalized over actin signal is indicated on top of each lane. **h**. Total spheroid areas for 4T1 cells silenced for *eIf5a1* (*n* = 8 4T1 no aspartate, *n* = 8 4T1 aspartate), *eIf5a2* (*n* = 10 4T1 no aspartate, *n* = 10 4T1 aspartate) or scramble shRNA (*n* = 9 4T1 no aspartate, *n* = 10 4T1 aspartate), grown in LLM supplemented with or without aspartate. Data are presented as mean ± s.d. (*n* indicates independent samples). Two-way ANOVA with Tukey’s multiple-comparison tests. **i**. Total spheroid areas for MCF10A H-Ras^v12^ cells silenced for *eIF5a1* (*n* = 12 MCF10A H-Ras^v12^ no aspartate, *n* = 11 MCF10A H-Ras^v12^ aspartate), *eIF5a2* (*n* = 11 MCF10A H-Ras^v12^ no aspartate, *n* = 10 MCF10A H-Ras^v12^ aspartate) or scramble shRNA (*n* = 10 MCF10A H-Ras^v12^ no aspartate, *n* = 11 MCF10A H-Ras^v12^ aspartate), grown in LLM supplemented with or without aspartate. Data are presented as mean ± s.d. (*n* indicates independent samples). Two-way ANOVA with Tukey’s multiple-comparison tests. **j**. ^13^C_4_-labeled fractions for various metabolites downstream of aspartate (included) in 4T1 spheroids (*n* = 3) grown in LLM supplemented with ^13^C_4_-aspartate. Data are presented as mean ± s.d. A representative graph of *n* = 3 experiments is shown. **k**. Average fractions of total carbon corresponding to ^13^C isotopes in different metabolites, in 4T1 spheroids (*n* = 3) grown in LLM supplemented with ^13^C_4_-aspartate. **l**. Histograms of CD44 surface expression in 4T1 spheroids dissociated with Trypsin (*n* = 3) or Accutase (*n* = 3). Data are normalized to mode (y axis is scaled so that the maximum of each curve is at 100) and further smoothed for display purposes. **m**. Intracellular *vs* cell surface levels of ^13^C-glutamate in 4T1 spheroids silenced for *Grin2d* (*n* = 3) or scramble shRNA (*n* = 3), grown in LLM supplemented with ^13^C_5_-glutamate. Data are presented as mean ± s.d. (*n* indicates independent samples). Two-way ANOVA with Tukey’s multiple-comparison tests. **n**. Total spheroid areas for 4T1 cells grown in LLM supplemented with or without L- or D-aspartate. *N* = 15 no aspartate, *n* = 16 L-aspartate, *n* = 13 D-aspartate. Data are presented as mean ± s.d. (*n* indicates independent samples). One-way ANOVA (*P* < 0.001) with Tukey’s multiple-comparison tests. Representative images are shown on the bottom. Scale bar = 250 μm. **o**. Hypusine levels in 4T1 spheroids grown in LLM supplemented with or without L- or D-aspartate. A representative image of *n* = 3 experiments is shown. Quantification of hypusine signal normalized over eIF5A signal is indicated on top of each lane. **p**. Hypusine levels in 4T1 spheroids grown in LLM supplemented with or without aspartate or glutamate. A representative image of *n* = 3 experiments is shown. Quantification of hypusine signal normalized over eIF5A signal is indicated on top of each lane. **q**. Grin2d detected in lung metastases 14 days after i.v. injections with 4T1 cancer cells, in mice pre-treated with daily injections of aspartate (20 mM, i.p., 10 days) or PBS, assessed by multiplex immunohistochemistry. Representative images from *n* = 5 4T1 shSCR PBS, *n* = 5 4T1 shSCR aspartate, *n* = 5 4T1 sh*Grin2d* PBS, *n* = 5 4T1 sh*Grin2d* aspartate independent mice are shown. Green = membrane marker ATP1A1; Red = Grin2d; Blue = DAPI nuclear staining. Scale bars = 25 μm. Arrows indicate membrane colocalization of Grin2d and ATP1A1. This staining is part of a multiplex staining and only relevant stains are shown. **r**. Relative fractions of *Grin2b* and *Grin2d* copies quantified by droplet digital PCR in 4T1 lung metastases, *n* = 3. Data are presented as mean ± s.d. (*n* indicates independent samples). Unpaired two-tailed *t*-test with Welch correction. **s**. Average ionomycin-normalized calcium response traces in 4T1 cells overexpressing *Grin2b*. The shaded ribbon represents standard error of the mean at each time point, while the grey rectangles indicate sequential addition of aspartate, glutamate and ionomycin at the times indicated by the arrows. A representative experiment (*n* = 38 cells) out of 2 independent experiments is shown. *Grin2b* overexpression stimulated a calcium response following glutamate but not aspartate addition in 4T1 cells. This shows that the NMDA receptor subunit expression determines the agonist to which breast cancer cells respond. **t**. Ionomycin-normalized calcium response upon sequential addition of aspartate and glutamate (*n* = 79 cells). Unpaired two-tailed *t*-test with Welch correction.

**Figure 2:**
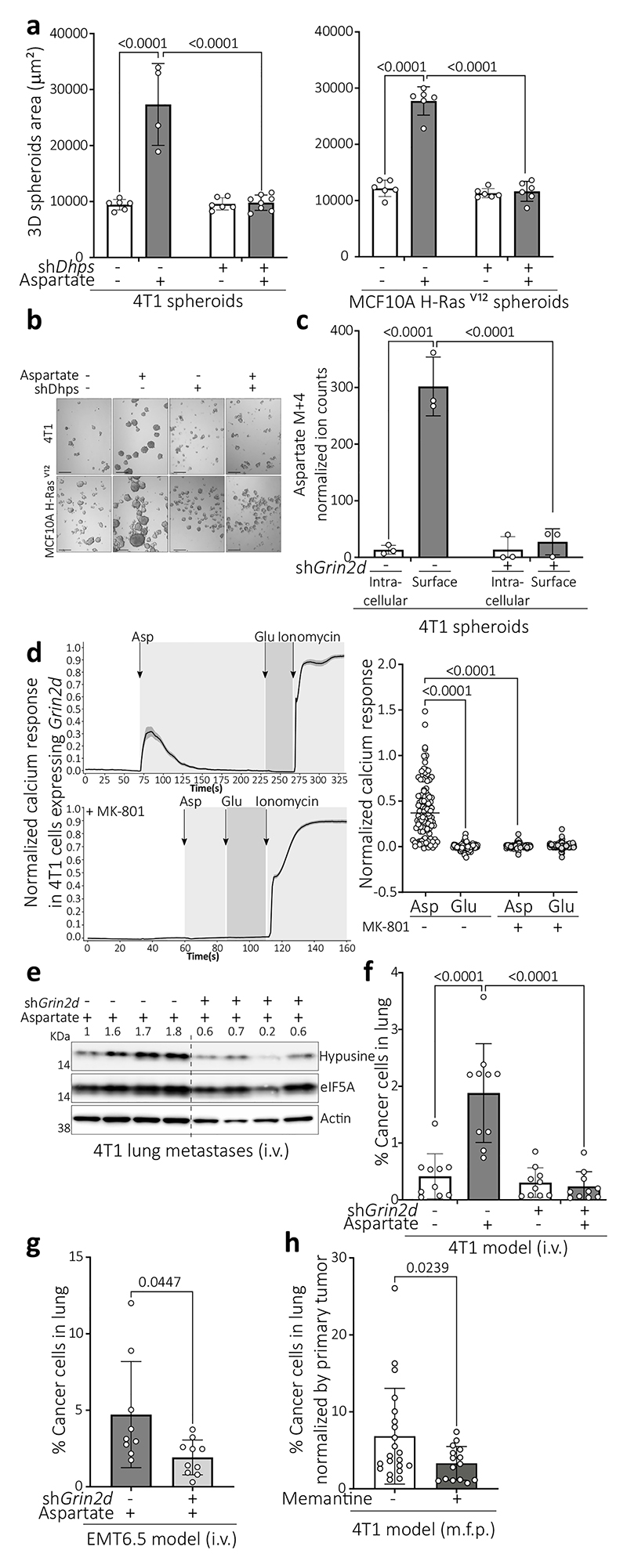
Aspartate triggers NMDA receptor activity **a**. Total spheroid areas for 4T1 (left) and MCF10A H-Ras^v12^ (right) cells silenced for *Dhps* (*n*=6 4T1 or H-Ras^v12^ no aspartate, *n*=8 4T1 aspartate, *n*=6 MCF10A H-Ras^v12^ aspartate) or scramble shRNA (*n*=6 4T1 or MCF10A H-Ras^v12^ no aspartate, *n*=4 4T1 aspartate, *n*=6 MCF10A H-Ras^v12^ aspartate) in lung-like medium (LLM) supplemented with aspartate. **b**. Representative images from **a**. Scale bar=250 μm. **c**. Intracellular *vs* cell surface levels of ^13^C-aspartate in 4T1 spheroids silenced for *Grin2d* or scramble shRNA (*n*=3 per group), supplemented with ^13^C_4_-aspartate in LLM. **d**. Average ionomycin-normalized calcium response traces with (bottom) or without (top) MK-801 treatment in 4T1 cells overexpressing *Grin2d* (left). Shaded ribbon: s.e.m., grey rectangles: sequential addition of aspartate/glutamate/ionomycin. One representative experiment (*n*=39 without MK-801 and *n*=73 with MK-801) out of 4 independent experiments is shown. Quantification of ionomycin-normalized calcium response in 4T1 cells overexpressing *Grin2d* without (*n*=106) or with (*n*=219) MK-801 treatment from 4 independent experiments (right). Two-way ANOVA with Šídák’s multiple-comparison tests considering all independent cells. **e**. Hypusine levels in 4T1 lung metastases silenced for *Grin2d* or scramble shRNA (*n*=4 per group) in mice pre-treated with aspartate or PBS. Hypusine quantification relative to total eIF5A shown on top of each lane. **f**. Percentage of CD90.1^+^ 4T1 cells silenced for *Grin2d* or scramble shRNA (*n*=10 per group) in mice pre-treated with aspartate or PBS. **g**. Percentage of CD90.1^+^ EMT6.5 cells silenced for *Grin2d* (*n*=10) or scramble shRNA (*n*=9) in mice pre-treated with aspartate. **h**. Percentage of CD90.1^+^ 4T1 cells in mice treated with Memantine-HCl (*n*=15) or PBS (*n*=21) normalized by primary tumor weight (g). For **a,c,f**, *P*-values based on Two-way ANOVA with Tukey’s multiple-comparison tests. For **g,h**, *P*-values based on unpaired two-tailed *t*-test with Welch correction. Data presented as mean±s.d. unless noted.

To assess our *in vivo* findings in cultured cells, we grew tumor spheroids from mouse 4T1 and human MCF10A H-Ras^V12^ breast cancer cells^6,9,10^, mouse B16F10 melanoma cells and human HUH7 hepatocellular carcinoma cells in medium resembling the glucose, amino acid, and organic acid concentrations of lung interstitial fluid and deprived or supplemented this media with 700 μM aspartate (lung-like medium, LLM; Supplementary Table 1). In line with our *in vivo* data, aspartate supplementation increased eIF5A hypusination and tumor spheroid growth (Extended Data Figure 2a-d). We verified increased overall translation and an enrichment of translation-related gene sets in MCF10A H-Ras^V12^ and/or 4T1 spheroids upon aspartate supplementation (Extended Data Figure 2e,f). *Dhps* silencing abrogated the aspartate-induced tumor spheroid growth (Figure 2a,b), which was phenocopied by *eIF5A1* and to some extent *eIF5A2* silencing (Extended Data Figure 2g-i). This shows that the LLM-based spheroid model recapitulates our *in vivo* findings.

## Aspartate promotes NMDA receptor activity

Next, we assessed the fate of aspartate in tumor spheroids with ^13^C tracer analysis^11^. When supplementing ^13^C_4_-aspartate to 4T1 tumor spheroids, we unexpectedly found that aspartate was not metabolized into its downstream products (Extended Data Figure 2j,k) suggesting that aspartate may bind to a cell surface protein. Therefore, we analyzed intracellular and cell surface aspartate separately (Extended Data Figure 2l) and found that most of the detected ^13^C_4_-aspartate was on the cell surface (Figure 2c). Therefore, we looked for receptors that can bind aspartate. The N-methyl-D-aspartate (NMDA) receptor, an ionotropic receptor of the brain activated by glutamate and glycine, can also bind aspartate, and the glutamate ionotropic receptor NMDA type subunit 2D (GRIN2D) is in biochemical assays the most responsive L- and D-aspartate binding subunit^12^. Therefore, we focused on GRIN2D. Indeed, *Grin2d* silencing significantly decreased the fraction of ^13^C_4_-aspartate, but not ^13^C_5_-glutamate, on the cell surface (Figure 2c, Extended Data Figure 2m). D-aspartate, but not glutamate, increased tumor spheroid growth and eIF5A hypusination (Extended Data Figure 2n-p). This non-responsiveness to glutamate may be explained by the observation that Grin2d was localized at the cell surface of cancer cells in 4T1 lung metastases (Extended Data Figure 2q), while *Grin2b*, encoding the classical glutamate-responding NMDA receptor subunit, was hardly expressed compared to *Grin2d* in 4T1 metastases (Extended Data Figure 2r). Next, we assessed NMDA receptor activity by measuring intracellular calcium response. We found that only aspartate, but not glutamate, stimulated a Grin2d-dependent calcium response, that was abrogated by the NMDA receptor inhibitor MK-801^13^ (Figure 2d, Supplementary Movie 1,2,3, Extended Data Figure 2s,t). Thus, we concluded that aspartate binds to Grin2d and activates the NMDA receptor in cancer cells.

## Hypusination depends on NMDA receptor activity

**Extended Data Figure 3:**
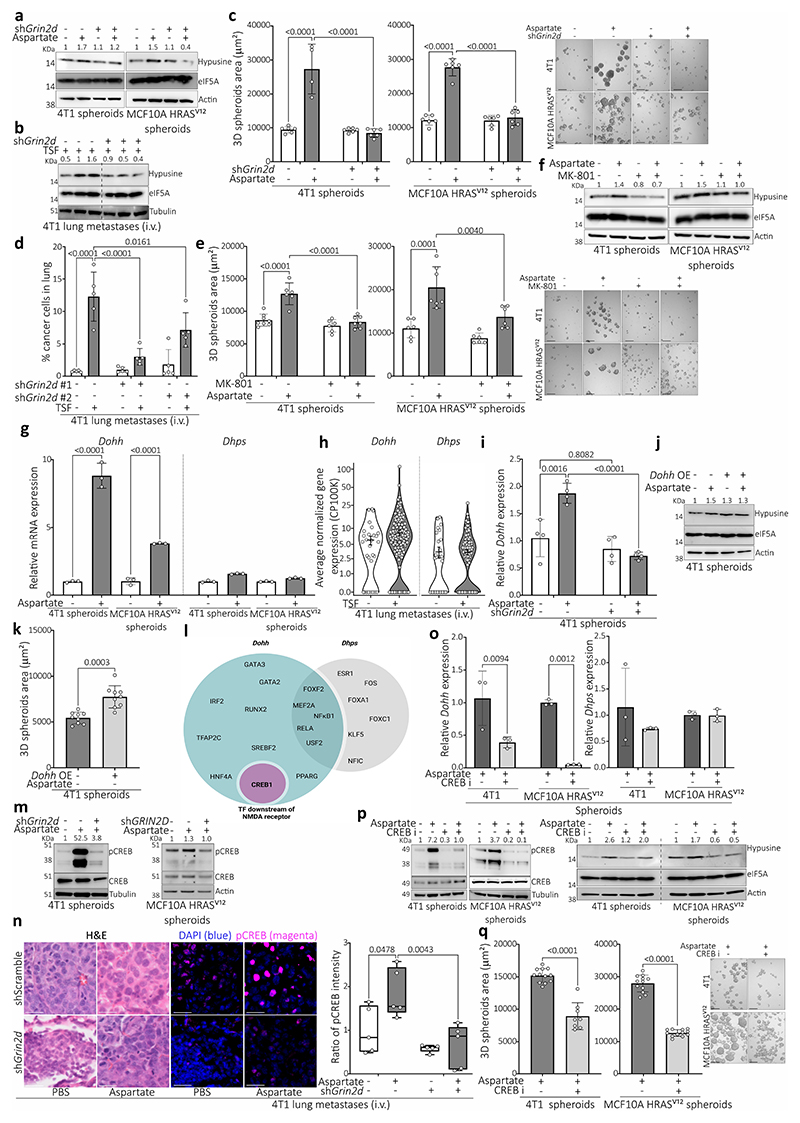
Aspartate-induced NMDA receptor activity promotes CREB phosphorylation **a**. Hypusine levels in 4T1 (left) and MCF10A H-Ras^v12^ (right) spheroids silenced for *Grin2d* or scramble shRNA, grown in lung-like medium (LLM) supplemented with or without aspartate. A representative image of *n* = 3 experiments is shown. Quantification of hypusine signal normalized over total eIF5A signal is indicated on top of each lane. **b**. Hypusine levels in 4T1 lung metastases silenced for *Grin2d* (*n* = 3) or scramble shRNA (*n* = 3) in mice pre-treated with tumor secreted factors (TSF). Quantification of hypusine signal normalized over eIF5A signal is indicated on top of each lane. **c**. Total spheroid areas for 4T1 (left) and MCF10A H-Ras^v12^ (right) cells silenced for *Grin2d* (*n* = 6 4T1 no aspartate, *n* = 6 MCF10A H-Ras^v12^ no aspartate, *n* = 6 4T1 aspartate, *n* = 6 MCF10A H-Ras^v12^ aspartate) or scramble shRNA (*n* = 6 4T1 no aspartate, *n* = 6 MCF10A H-Ras^v12^ no aspartate, *n* = 4 4T1 aspartate, *n* = 6 MCF10A H-Ras^v12^ aspartate), grown in LLM supplemented with or without aspartate. Data are presented as mean ± s.d. (*n* indicates independent samples). Two-way ANOVA with Tukey’s multiple-comparison tests. Representative images are shown on the right. Scale bar = 250 μm. Data from the scramble group are the same as those in the scramble group from Figure 2a. **d**. Percentage of cancer cells present in the lung 16 days after i.v. injections with CD90.1^+^ 4T1 cells silenced for *Grin2d* (with two different shRNA sequences) or scramble shRNA in mice pre-treated with TSF or control medium (*n* = 5 4T1 shSCR control medium, *n* = 5 4T1 shSCR TSF, *n* = 5 4T1 sh*Grin2d*#1 control medium, *n* = 5 4T1 sh*Grin2d*#1 TSF, *n* = 5 4T1 sh*Grin2d*#2 control medium, *n* = 5 4T1 sh*Grin2d*#2 TSF). Data are presented as mean ± sd. Two-way ANOVA with Šídák’s multiple-comparison tests. **e**. Total spheroid areas for 4T1 (left) and MCF10A H-Ras^v12^ (right) cells grown in LLM supplemented with or without aspartate and treated with or without the NMDA receptor inhibitor MK-801 for 3 days. *N* = 8 4T1 no aspartate, *n* = 6 4T1 aspartate, *n* = 6 4T1 no aspartate with MK-801, *n* = 7 4T1 aspartate with MK-801, *n* = 6 MCF10A H-Ras^v12^ no aspartate, *n* = 7 MCF10A H-Ras^v12^ aspartate, *n* = 6 MCF10A H-Ras^v12^ no aspartate with MK-801, *n* = 6 MCF10A H-Ras^v12^ aspartate with MK-801. Data are presented as mean ± s.d. (*n* indicates independent samples). Representative images are shown on the right. Scale bar = 250 μm. Two-way ANOVA with Tukey’s multiple-comparison tests. **f**. Hypusine levels in 4T1 (left) and MCF10A H-Ras^v12^ (right) spheroids grown in LLM supplemented with or without aspartate and treated with or without the NMDA receptor inhibitor MK-801 for 3 days. A representative image of *n* = 3 independent experiments is shown. Quantification of hypusine signal normalized over eIF5A signal is indicated on top of each lane. **g**. Relative mRNA expression of *Dohh* and *Dhps* in 4T1 and MCF10A H-Ras^v12^ spheroids grown in LLM supplemented with or without aspartate. Data for each gene and cell line are normalized relative to the average of the respective control (no aspartate) condition. Bars represent averages, and single dots individual replicates. Error bars represent ± s.d. (*n* = 3 independent replicates). Three-way ANOVA with Tukey’s multiple-comparison tests. **h**. *Dohh* and *Dhps* mRNA expression levels based on scRNA-seq data for cancer cells in 4T1 lung metastases from mice pre-treated with TSF (*n* = 139 cells) or control medium (*n* = 29 cells). Data are normalized as counts per 100k reads (CP100K). Crossbars represent mean ± s.e.m. **i**. Relative mRNA expression of *Dohh* in 4T1 spheroids silenced for *Grin2d* or scramble shRNA, grown in LLM supplemented with or without aspartate. Data are normalized relative to the average of the respective control (scramble no aspartate) condition. Bars represent averages, and single dots individual replicates. Error bars represent ± s.d. (*n* = 4 independent replicates). Two-way ANOVA with Šídák’s multiple-comparison tests. **j**. Hypusine levels in 4T1 spheroids overexpressing *Dohh* or an empty vector, grown in LLM supplemented with or without aspartate. A representative image of *n* = 3 experiments is shown. Quantification of hypusine signal normalized over total eIF5A signal is indicated on top of each lane. **k**. Total spheroid areas for 4T1 cells overexpressing *Dohh* (*n* = 9) or an empty vector (*n* = 9), grown in LLM supplemented without aspartate for 3 days. Data are presented as mean ± s.d. (*n* indicates independent samples). Unpaired two-tailed *t*-test with Welch correction. **l**. Venn diagram depicting the intersection between transcription factors downstream of NMDA receptor and predicted to bind to *Dohh* or *Dhps*. Prediction was based on the JASPAR Predicted Transcription Factor Targets from the Harmonizome database (maayanlab.cloud/Harmonizome/dataset). **m**. Phosphorylated CREB levels in 4T1 (left) and MCF10A H-Ras^v12^ (right) spheroids silenced for *Grin2d* or scramble shRNA, grown in LLM supplemented with or without aspartate. A representative image of *n* = 3 experiments is shown. Quantification of phosphorylated CREB signal normalized over total CREB signal is indicated on top of each lane. **n**. Left: representative H&E staining for 4T1 lung metastases represented in Figure 3a, Figure 4a,c, Extended Data Figure 3n. Middle: phosphorylated CREB detected in lung metastases 14 days after i.v. injection with 4T1 cancer cells, in mice pre-treated with daily injections of aspartate (20 mM, i.p., 10 days) or PBS, assessed by multiplex immunohistochemistry. Representative images from *n* = 5 4T1 shSCR PBS, *n* = 5 4T1 shSCR aspartate, *n* = 5 4T1 sh*Grin2d* PBS, *n* = 5 4T1 sh*Grin2d* aspartate independent mice are shown. Blue = DAPI nuclear staining; magenta = pCREB. Scale bars = 25 μm. Right: ratio of pCREB intensity normalized on 4T1 shSCR PBS. Box hinges indicate the 1^st^/3^rd^ quartiles of the corresponding data, while box mid-lines represent medians, and whiskers span the range of the data. Individual data points are indicated by the white dots. Two-way ANOVA with Tukey’s multiple-comparison tests. This staining is part of a multiplex staining and only relevant stains are shown. Stainings for H&E and pCREB orginate from consecutive cuts. The same metastatic regions are also used in Figure 3a and Figure 4a,c. **o**. Relative mRNA expression of *Dohh* (left) and *Dhps* (right) in 4T1 and MCF10A H-Ras^v12^ spheroids grown in LLM supplemented with aspartate and treated with or without the CREB inhibitor Compound3i. Data for each gene are normalized relative to the average of the respective control (no CREB inhibitor) condition. Bars represent averages, and single dots individual replicates. Error bars represent ± s.d. (*n* = 3 independent replicates). Two-way ANOVA with Šídák’s multiple-comparison tests. **p**. Phosphorylated CREB (left) and hypusine (right) levels in 4T1 and MCF10A H-Ras^v12^ spheroids grown in LLM supplemented with aspartate and treated with or without the CREB inhibitor Compound3i. A representative image of *n* = 3 experiments is shown. Quantification of phosphorylated CREB signal normalized over total CREB signal or hypusine signal normalized over total eIF5A signal is indicated on top of each lane. **q**. Total spheroid areas for 4T1 (left) and MCF10A H-Ras^v12^ (right) cells grown in LLM supplemented with aspartate and treated with or without the CREB inhibitor Compound3i. *N* = 12 4T1 aspartate, *n* = 8 4T1 aspartate with CREB inhibitor, *n* = 12 MCF10A H-Ras^v12^ aspartate, *n* = 12 MCF10A H-Ras^v12^ aspartate with CREB inhibitor. Data are presented as mean ± s.d. (*n* indicates independent samples). Unpaired two-tailed *t*-test with Welch correction. Representative images are shown on the right. Scale bar = 250 μm.

Next, we asked whether the NMDA receptor activity regulates eIF5A hypusination. In this line, *Grin2d* silencing decreased aspartate- or TSF-induced hypusination in tumor spheroids and/or lung metastases (Figure 2e, Extended Data Figure 3a, b) as well as tumor spheroid (4T1, MCF10A H-Ras^V12^) and/or lung metastasis (4T1 and EMT6.5) growth (Figure 2f, g, Extended Data Figure 3c, d). The same result was observed upon treatment with MK-801 (Extended Data Figure 3e, f) or the clinically approved NMDA receptor inhibitor memantine^13^ (Figure 2h). Thus, we conclude that the activation of the NMDA receptor by aspartate induces eIF5A hypusination and lung metastasis aggressiveness.

## NMDA receptor activity increases DOHH

**Figure 3:**
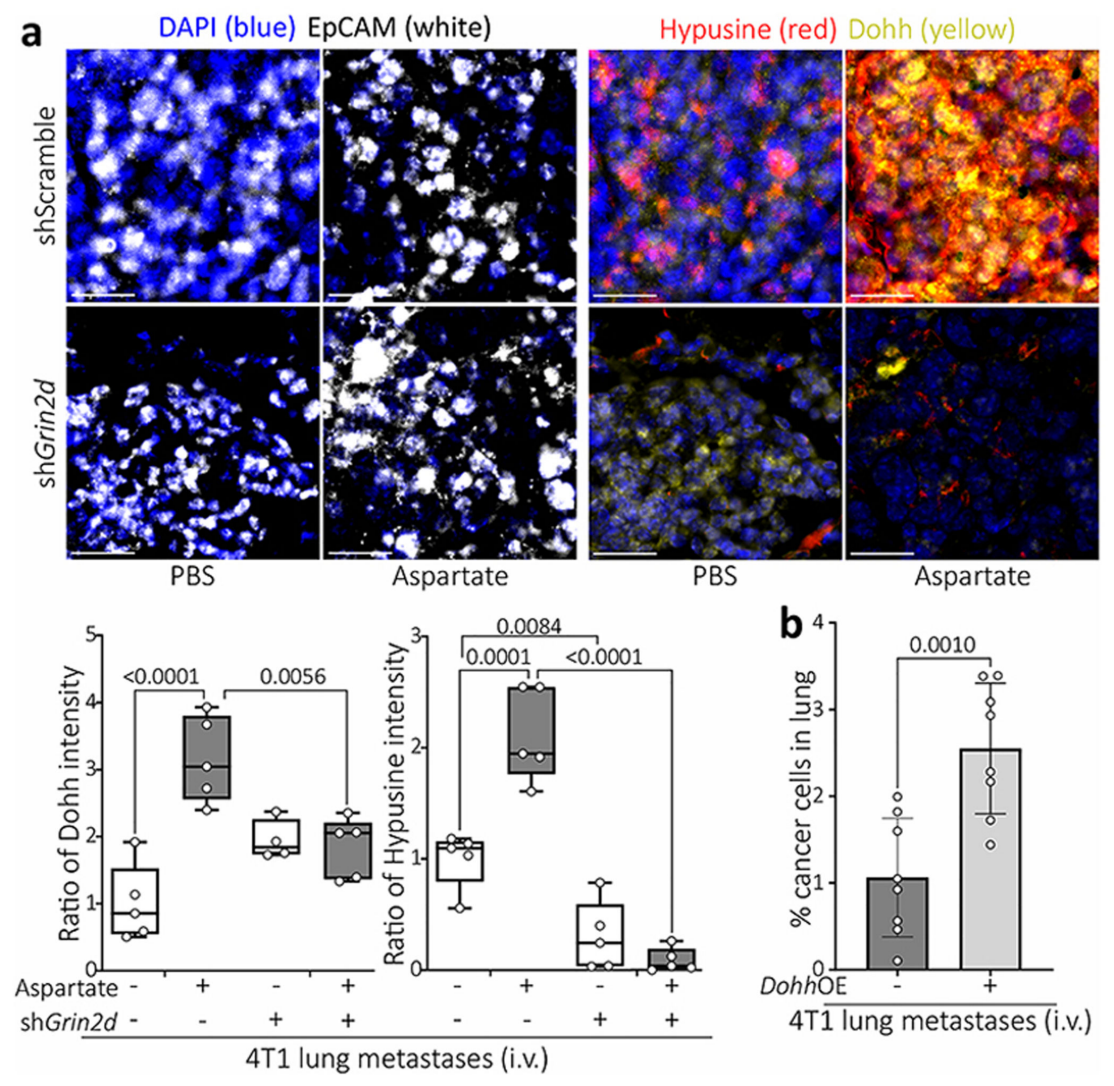
Aspartate-induced NMDA receptor activity regulates DOHH expression **a**. Hypusine and Dohh detected in 4T1 metastases silenced for *Grin2d* or scramble shRNA in mice pre-treated with aspartate or PBS. Top: representative images from *n* = 5 independent mice per group are shown. White = EpCAM; blue = DAPI nuclear staining; red = Hypusine; yellow = Dohh. Scale bars = 25 μm. Bottom: ratio of Dohh (left) and Hypusine (right) intensities normalized on 4T1 shSCR PBS. Box hinges indicate the 1^st^/3^rd^ quartiles of the corresponding data, while box mid-lines represent medians, and whiskers span the range of the data. Individual data points are indicated by the white dots. Two-way ANOVA with Tukey’s multiple-comparison tests. This staining is part of a multiplex staining and only relevant stains are shown, the same metastatic regions and Hypusine stainings are also used in Figure 4a,c and Extended Data Figure 3n. **b**. Percentage of CD90.1^+^ 4T1 cells overexpressing *Dohh* or an empty vector (*n* = 8 per group) in mouse lungs. Data are presented as mean ± sd. Unpaired two-tailed *t*-test with Welch correction.

Next, we investigated how NMDA receptor activity increases eIF5A hypusination. The activity of DHPS and deoxyhypusine hydroxylase (DOHH) is required for hypusination (Extended Data Figure 1h). We observed that aspartate increased *Dohh*, but not *Dhps*, expression in tumor spheroids (Extended Data Figure 3g) and the same was observed in lung metastases from mice treated with TSF (Extended Data Figure 3h). Moreover, the aspartate induced increase in DOHH expression was abrogated by *Grin2d* silencing in tumor spheroids (Extended Data Figure 3i). Accordingly, DOHH and hypusine were co-expressed in lung metastases of aspartate pre-treated mice compared to control or *Grin2d* silenced metastases (Figure 3a). Importantly, overexpression *of Dohh* was sufficient to increase eIF5A hypusination and tumor spheroid growth in the absence of aspartate (Extended Data Figure 3j,k) and metastasis aggressiveness in control mice (Figure 3b). Thus, this demonstrates that increased *DOHH* expression is sufficient to promote eIF5A hypusination resulting in more aggressiveness lung metastases.

Next, we asked how NMDA receptor activity induces DOHH expression. We computationally predicted potential transcription factors regulating *DOHH* using the Harmonizome database^14^. Out of the predicted transcription factors only cAMP response element-binding protein (CREB) was known to be regulated by NMDA receptor activity^15^ (Extended Data Figure 3l). In this line, we observed that aspartate induced CREB phosphorylation, which decreased upon *Grin2d* silencing in tumor spheroids and lung metastases (Extended Data Figure 3m, n). Moreover, inhibition of CREB with Compound 3i^16^ decreased *Dohh* (but not *Dhps*) expression, eIF5A hypusination and tumor spheroid growth (Extended Data Figure 3o-q). Thus, we concluded that aspartate-induced NMDA receptor activity results in CREB phosphorylation driving increased *DOHH* expression.

## eIF5A-induced translation increases collagen

**Extended Data Figure 4:**
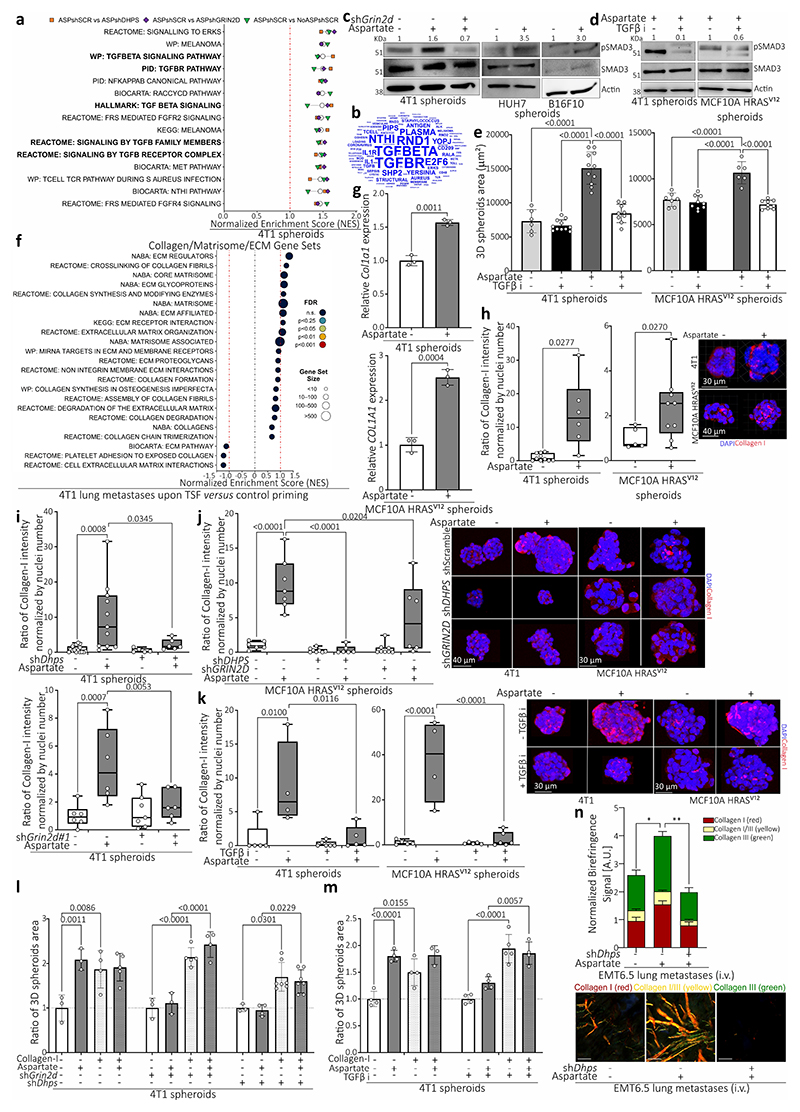
eIF5A hypusination results in TGF-β-mediated collagen synthesis **a**. GSEA normalized enrichment scores (NES) for the top 15 gene sets commonly upregulated in translation based on changes in the ratio of Polysomal to Subpolysomal RNA levels, between 4T1 spheroids silenced for scramble shRNA grown in lung-like medium (LLM) supplemented with aspartate and 4T1 spheroids silenced for (i) *Dhps* (orange symbols) or (ii) *Grin2d* (purple symbols) grown in LLM supplemented with aspartate, or (iii) 4T1 spheroids silenced for scramble shRNA grown in LLM without aspartate (green symbols). White dots represent the average NES over all three comparisons. Gene sets related to TGF-β signaling are highlighted in bold. **b**. Word cloud highlighting the top 100 most frequently found terms among enriched gene sets, based on GSEA results analogous to those used to generate Extended Data Figure 4a, but considering all three comparisons simultaneously (see *Methods*). **c**. Phosphorylated SMAD3 levels in 4T1 spheroids silenced for *Grin2d* or scramble shRNA (left), HUH7 (middle) and B16F10 (right) spheroids, grown in LLM supplemented with or without aspartate. A representative image of *n* = 3 experiments is shown. Quantification of phosphorylated SMAD3 signal normalized over total SMAD3 signal is indicated on top of each lane. **d**. Phosphorylated SMAD3 levels in 4T1 (left) and MCF10A H-Ras^v12^ (right) spheroids grown in LLM supplemented with aspartate and treated with or without a TGF-β inhibitor. A representative image of *n* = 3 experiments is shown. Quantification of phosphorylated SMAD3 signal normalized over total SMAD3 signal is indicated on top of each lane. **e**. Total spheroid areas for 4T1 (left) and MCF10A H-Ras^v12^ (right) cells grown in LLM supplemented with or without aspartate and treated with or without the TGF-β inhibitor for 5 (4T1) or 3 days (MCF10A H-Ras^v12^). *N* = 6 4T1 no aspartate, *n* = 11 4T1 no aspartate with TGF-β inhibitor, *n* = 11 4T1 aspartate, *n* = 9 aspartate with TGF-β inhibitor; *n* = 7 MCF10A H-Ras^v12^ no aspartate, *n* = 6 MCF10A H-Ras^v12^ aspartate, *n* = 9 MCF10A H-Ras^v12^ no aspartate with TGF-β inhibitor, *n* = 9 MCF10A H-Ras^v12^ aspartate with TGF-β inhibitor. Data are presented as mean ± s.d. (*n* indicates independent samples). Two-way ANOVA with Tukey’s multiple-comparison tests. **f**. GSEA normalized enrichment scores (NES) for gene sets containing either of the terms COLLAGEN, MATRISOME, ECM, and EXTRACELLULAR_MATRIX found on cancer cells based on scRNA-seq comparing 4T1 lung metastases from mice pre-treated with control medium or TSF. Dot colors and areas indicate FDR-adjusted *P*-values and gene-set sized, respectively. *P-*values based on *fgsea*’s adaptive multilevel splitting Monte Carlo approach, subject to FDR adjustment using the Benjamini-Hochberg (BH) approach. **g**. Relative mRNA expression of *Col1a1* in 4T1 (top) and MCF10A H-Ras^v12^ (bottom) spheroids grown in LLM supplemented with or without aspartate. Data for each gene and cell line are normalized relative to the average of the respective control (no aspartate) condition. Bars represent averages, and single dots individual replicates. Error bars represent ± s.d. (*n* = 3 independent replicates). Unpaired two-tailed *t*-test with Welch correction. **h**. Box and whisker plots of relative abundance of collagen I in 4T1 (left) and MCF10A H-Ras^v12^ (right) spheroids grown in LLM supplemented with or without aspartate, measured by immunofluorescence. *N* = 10 4T1 no aspartate, *n* = 6 4T1 aspartate, and *n* = 5 MCF10A H-Ras^v12^ no aspartate and *n* = 9 MCF10A H-Ras^v12^ aspartate. The total fluorescence intensity was normalized over the number of DAPI-stained nuclei. Relative fluorescence intensities per cell are depicted, normalized to the mean intensity for the control condition. Box hinges indicate the 1^st^/3^rd^ quartiles of the corresponding data, while box mid-lines represent medians, and whiskers span the range of the data. Individual data points are indicated by the white dots. Unpaired two-tailed *t*-test with Welch correction. Representative three-dimensional representation are depicted on the right. Blue, DAPI-stained nuclei; red, collagen I. **i**. Box and whisker plots of relative abundance of collagen I in 4T1 spheroids silenced for *Dhps* (top) or *Grin2d* (bottom) or scramble shRNA, grown in LLM supplemented with or without aspartate, measured by immunofluorescence. Top: *n* = 15 no aspartate scramble, *n* = 11 aspartate scramble, *n* = 5 no aspartate sh*Dhps, n* = 6 aspartate sh*Dhps*. Bottom: *n* = 6 no aspartate scramble, *n* = 6 aspartate scramble, *n* = 7 no aspartate sh*Grin2d, n* = 6 aspartate sh*Grin2d*. The total fluorescence intensity was normalized over the number of DAPI-stained nuclei. Relative fluorescence intensities per cell are depicted, normalized to the mean intensity for the control condition. Box hinges indicate the 1^st^/3^rd^ quartiles of the corresponding data, while box mid-lines represent medians, and whiskers span the range of the data. Individual data points are indicated by the white dots. Two-way ANOVA with Tukey’s multiple-comparison tests. Representative three-dimensional representation are depicted in Extended Data Figure 4j. **j**. Box and whisker plots of relative abundance of collagen I in MCF10A H-Ras^v12^ spheroids silenced for *DHPS, GRIN2D* or scramble shRNA, grown in LLM supplemented with or without aspartate, measured by immunofluorescence. *N* = 6 no aspartate scramble, *n* = 7 aspartate scramble, *n* = 8 no aspartate sh*DHPS, n* = 6 aspartate sh*DHPS, n* = 8 no aspartate sh*GRIN2D, n* = 6 aspartate sh*GRIN2D*. The total fluorescence intensity was normalized over the number of DAPI-stained nuclei. Relative fluorescence intensities per cell are depicted, normalized to the mean intensity for the control condition. Box hinges indicate the 1^st^/3^rd^ quartiles of the corresponding data, while box mid-lines represent medians, and whiskers span the range of the data. Individual data points are indicated by the white dots. Two-way ANOVA with Tukey’s multiple-comparison tests. Representative three-dimensional representation are depicted on the right and include samples shown in Extended Data Figure 4i. Blue, DAPI-stained nuclei; red, collagen I. **k**. Box and whisker plots of relative abundance of collagen I in 4T1 (left) and MCF10A H-Ras^v12^ (right) spheroids grown in LLM supplemented with or without aspartate and treated with or without the TGF-β inhibitor, measured by immunofluorescence. *N* = 5 4T1 no aspartate, *n* = 4 4T1 aspartate, *n* = 6 4T1 no aspartate with TGF-β inhibitor, *n* = 5 4T1 no aspartate with TGF-β inhibitor, and *n* = 5 MCF10A H-Ras^v12^ no aspartate, *n* = 4 MCF10A H-Ras^v12^ aspartate, *n* = 5 MCF10A H-Ras^v12^ no aspartate with TGF-β inhibitor, *n* = 6 MCF10A H-Ras^v12^ aspartate with TGF-β inhibitor. The total fluorescence intensity was normalized over the number of DAPI-stained nuclei. Relative fluorescence intensities per cell are depicted, normalized to the mean intensity for the control condition. Box hinges indicate the 1^st^/3^rd^ quartiles of the corresponding data, while box mid-lines represent medians, and whiskers span the range of the data. Individual data points are indicated by the white dots. Two-way ANOVA with Šídák’s multiple-comparison tests. Representative three-dimensional representation are depicted on the right. Blue, DAPI-stained nuclei; red, collagen I. **l**. Relative spheroid areas for 4T1 cells silenced for *Grin2d, Dhps* or scramble shRNA grown in LLM with 1.5% Matrigel, supplemented with or without aspartate and with or without 1.5% Collagen-I. *N* = 3 no aspartate scramble, *n* = 3 aspartate scramble, *n* = 4 no aspartate scramble with Collagen-I, *n* = 5 aspartate scramble with Collagen-I; *n* = 3 no aspartate sh*Dhps, n* = 3 aspartate sh*Dhps, n* = 5 no aspartate sh*Dhps* with Collagen-I, *n* = 4 aspartate sh*Dhps* with Collagen-I; *n* = 3 no aspartate sh*Grin2d, n* = 4 aspartate sh*Grin2d, n* = 7 no aspartate sh*Grin2d* with Collagen-I, *n* = 7 aspartate sh*Grin2d* with Collagen-I. Data are presented as mean ± s.d, normalized to the mean spheroid areas for the respective no aspartate conditions (*n* indicates independent samples). Two-way ANOVA with Tukey’s multiple-comparison tests. **m**. Relative spheroid areas for 4T1 cells grown in LLM with 1.5% Matrigel, supplemented with or without aspartate and with or without 1.5% Collagen-I and treated with or without the TGF-β inhibitor. *N* = 4 no aspartate, *n* = 4 aspartate, *n* = 4 no aspartate with Collagen-I, *n* = 4 aspartate with Collagen-I; *n* = 4 no aspartate with TGF-β inhibitor, *n* = 4 aspartate with TGF-β inhibitor, *n* = 5 no aspartate with TGF-β inhibitor and Collagen-I, *n* = 4 aspartate with TGF-β inhibitor and Collagen-I. Data are presented as mean ± s.d, normalized to the mean spheroid area for the respective no aspartate conditions (*n* indicates independent samples). Two-way ANOVA with Tukey’s multiple-comparison tests. **n**. Top: Quantification of linearized collagen based on Picrosirius Red staining and polarized light microscopy detected in EMT6.5 lung metastases silenced for *Dhps* or scramble shRNA in mice pre-treated with daily injections of aspartate (20 mM, i.p., 10 days) or PBS. Significant collagen red/yellow/green increase (*) 0.0023/0.1758/0.0042 EMT6.5 aspartate scramble, *n* = 5 PBS and *n* = 5 aspartate. Significant collagen red/yellow/green decrease (**) <0.0001/<0.0001/<0.0001 (EMT6.5 sh*Dhps*), *n* = 5 PBS scramble, *n* = 5 aspartate scramble and *n* = 5 aspartate sh*Dhps*. Data are normalized by metastasis area (megapixel) and expressed as mean ± SEM. Two-way ANOVA with Šídák’s multiple-comparison tests. Bottom: representative images of linearized collagen based on Picrosirius Red staining and polarized light microscopy detected in EMT6.5 lung metastases. Red color mostly indicates thick collagen I fibers and green color mostly indicates thin collagen III fibers. A representative image of *n* = 5 EMT6.5 PBS scramble, *n* = 5 EMT6.5 aspartate scramble, *n* = 5 EMT6.5 aspartate sh*Dhps* lung metastases is shown. Scale bars = 20 μm.

**Figure 4:**
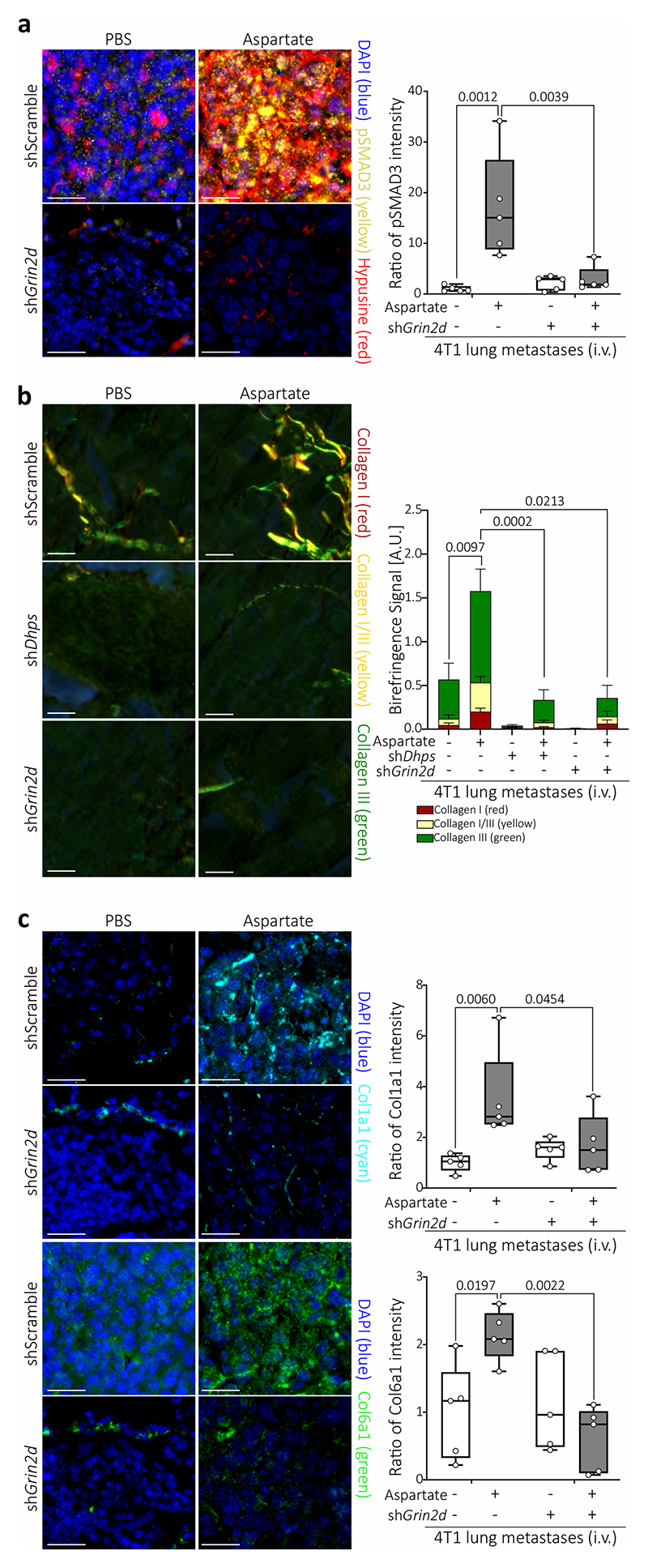
Alternative translation induced by eIF5A hypusination results in collagen synthesis **a**. Phosphorylated SMAD3 and Hypusine in 4T1 metastases silenced for *Grin2d* or scramble shRNA in mice pre-treated with aspartate or PBS. Left: representative images from *n* = 5 independent mice per group. Blue=DAPI nuclear staining; yellow=pSMAD3; red=Hypusine. Scale bars=25 μm. Right: ratio of pSMAD3 intensity normalized on 4T1 shSCR PBS. Two-way ANOVA with Tukey’s multiple-comparison tests. **b**. Left: representative images of linearized collagen detected in 4T1 metastases silenced for *Grin2d, Dhps* or scramble shRNA in mice pre-treated with aspartate or PBS. Red color mostly indicates thick collagen I fibers and green color mostly indicates thin collagen III fibers. Scale bars=10 μm. Right: quantification of linearized collagen, significant collagen green increase (*P*=0.0097) compared to 4T1 aspartate scramble; significant collagen green decrease (*P*=0.0002) compared to 4T1 aspartate sh*Dhps* and (*P*=0.0213) 4T1 aspartate sh*Grin2d. N*=4 PBS and aspartate scramble, *n*=4 PBS sh*Dhps, n*=5 aspartate sh*Dhps, n*=3 PBS sh*Grin2d*, and *n*=4 aspartate sh*Grin2d*. Data are expressed as mean ± SEM. Two-way ANOVA with Šídák’s multiple-comparison tests. **c**. Col1a1 and Col6a1 detected in 4T1 metastases silenced for *Grin2d* or scramble shRNA in mice pre-treated with aspartate or PBS. Left: representative images of *n*=5 independent mice per group. Cyan=Col1a1; green=Col6a1; blue=DAPI nuclear staining. Scale bars=25 μm. Right: ratio of Col1a1 (top) and Col6a1 (bottom) intensities normalized on 4T1 shSCR PBS. Two-way ANOVA with Dunnett’s multiple-comparison tests. Stainings for Col1a1 and Col6a1 originate from consecutive cuts. For **a,c**, box hinges indicate 1^st^/3^rd^ quartiles of the corresponding data, while box mid-lines represent medians, and whiskers span the range of the data. White dots indicates individual data points. The staining shown in **a,c** are part of a multiplex staining and only relevant stains are shown, the same metastatic regions and Hypusine stainings are also used in Figure 3a and Extended Data Figure 3n.

Next, we addressed which translational program is activated downstream of eIF5A hypusination using polysome profiling. We identified multiple signatures indicative of TGF-β signaling among the highest ranking gene sets regulated by aspartate and decreased upon *Dhps* and *Grin2d* silencing (Extended Data Figure 4a,b), which was functionally confirmed by analyzing the phosphorylation of the canonical TGF-β signaling target SMAD3^17^ (Extended Data Figure 4c). Moreover, inhibition of TGF-β with LY364947 decreased aspartate-induced SMAD3 phosphorylation and tumor spheroid growth (Extended Data Figure 4d-e). Accordingly, lung metastases from aspartate pre-treated mice displayed a co-expression of hypusine and phosphorylated SMAD3 compared to control and *Grin2d* silenced metastases (Figure 4a). Thus, we concluded that eIF5A hypusination increases canonical TGF-β signaling. Next, we asked how TGF-β signaling enables the aggressiveness of lung metastases. We focused on collagen synthesis which is known to be regulated by TGF-β signaling^17^ and to promote lung metastasis^10^. In line, we found a signature indicative of collagen remodeling in lung metastases from TSF treated mice (Extended Data Figure 4f). Moreover, we detected increased *COL1A1* expression and collagen-I abundance in aspartate treated tumor spheroids (Extended Data Figure 4g,h), which decreased upon *Dhps* and *Grin2d* silencing well as inhibition of TGF-β signaling (Extended Data Figure 4i-k). In addition, we observed that collagen-I supplementation rescued the impaired the growth of *Dhps* and *Grin2d* silenced as well as TGF-β inhibitor treated 4T1 spheroids (Extended Data Figure 4l,m). Accordingly, aggressive metastases from mice pre-treated with aspartate showed an increased abundance of collagen I-III as well as COL1A1 and COL6A1 compared to control (Figure 4b,c, Extended Data Figure 4n), which was abrogated by *Dhps* and *Grin2d* silencing (Figure 4b,c, Extended Data Figure 4n). Thus, we show that the translational program induced by eIF5A hypusination converges via TGF-β signaling into metastasis promoting collagen synthesis by cancer cells.

## Evidence of aspartate signaling in patients

**Extended Data Figure 5:**
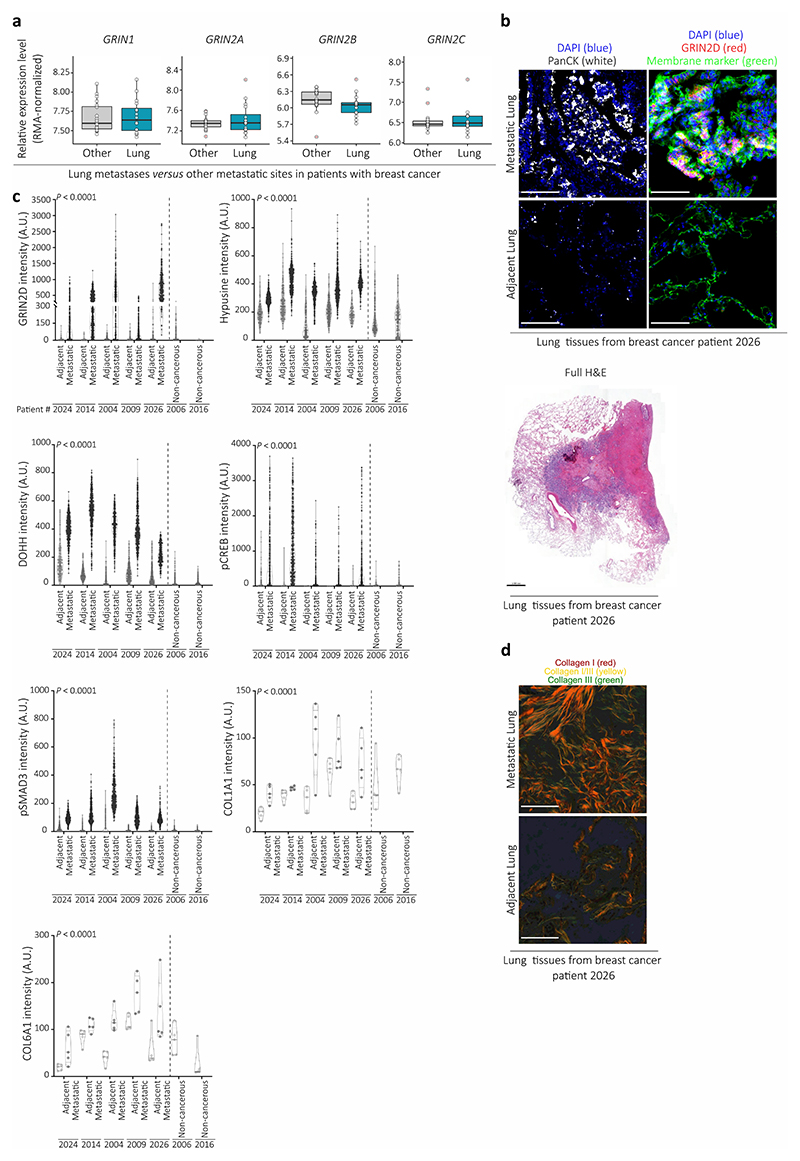
Expression of NMDA receptor subunits and staining of proteins indicative of aspartate signaling in patients with breast cancer **a**. RMA-normalized mRNA expression levels of *NMDA-*receptor-related genes in breast cancer-derived lung metastases *vs* breast cancer-derived bone/brain/liver metastases, based on patient biopsies (GSE14018). *N* = 16 lung metastases and *n* = 20 non-lung metastases samples (*n* = 8 bone metastases, *n* = 7 brain metastases, *n* = 5 liver metastases). Box hinges indicate the 1^st^/3^rd^ quartiles of the corresponding data, while box mid-lines represent medians, and whiskers span the range of the data excluding outliers (data points more than 1.5 x IQR away from the 1^st^/3^rd^ quartiles, where IQR = inter-quartile range), with the latter indicated in red. Individual data points are indicated by the white dots. Statistics show *P*-values based on differential expression analysis with *limma*. **b**. Top: DAPI and PanCK (left), GRIN2D and membrane marker (right) detected in metastatic and adjacent lungs from the UPTIDER breast cancer patient 2026 shown in Figure 5e, assessed by multiplex immunohistochemistry. Blue = DAPI nuclear staining, red = GRIN2D, green = membrane marker ATP1A1 (scale bars = 100 μm). Membrane marker has been added to identify surface localization of GRIN2D, the same exact representative image was used in Figure 5e. Bottom: representative image of H&E staining of the whole lung from patient 2026, scale bar = 2 mm. H&E and multiplex immunohistochemistry stainings originate from non-consecutive cuts. **c**. Quantification of GRIN2D, Hypusine, DOHH, nuclear pCREB, nuclear pSMAD3, COL1A1 and COL6A1 intensities in tissues derived from the UPTIDER breast cancer patients. For GRIN2D, Hypusine and DOHH, fluorescence intensity values per single cell are depicted, across 5 independent regions. For pCREB and pSMAD3, fluorescence intensity values per nucleus are depicted, across 5 independent regions. For COL1A1 and COL6A1, mean fluorescence intensities per unit area are depicted, across 5 independent regions. Two-way ANOVA, with *P* values for the tissue type (metastatic *vs* adjacent) factor shown above the graphs. All single comparisons per patients were significant based on unpaired two-tailed *t*-test with Welch correction, except for COL1A1 and COL6A1, for which single comparisons per patients were significant in 7 out of 10 measurements based on unpaired two-tailed *t*-test with Welch correction. Representative images are shown in Figure 5e and Extended Data Figure 6. **d**. Representative images of linearized collagen based on Picrosirius Red staining and polarized light microscopy detected in metastatic and adjacent lungs from the UPTIDER breast cancer patient 2026, quantified in Figure 5f. Red color mostly indicates thick collagen I fibers and green color mostly indicates thin collagen III fibers. A representative image of *n* = 5 patients is shown. Scale bars = 1 mm.

**Extended Data Figure 6:**
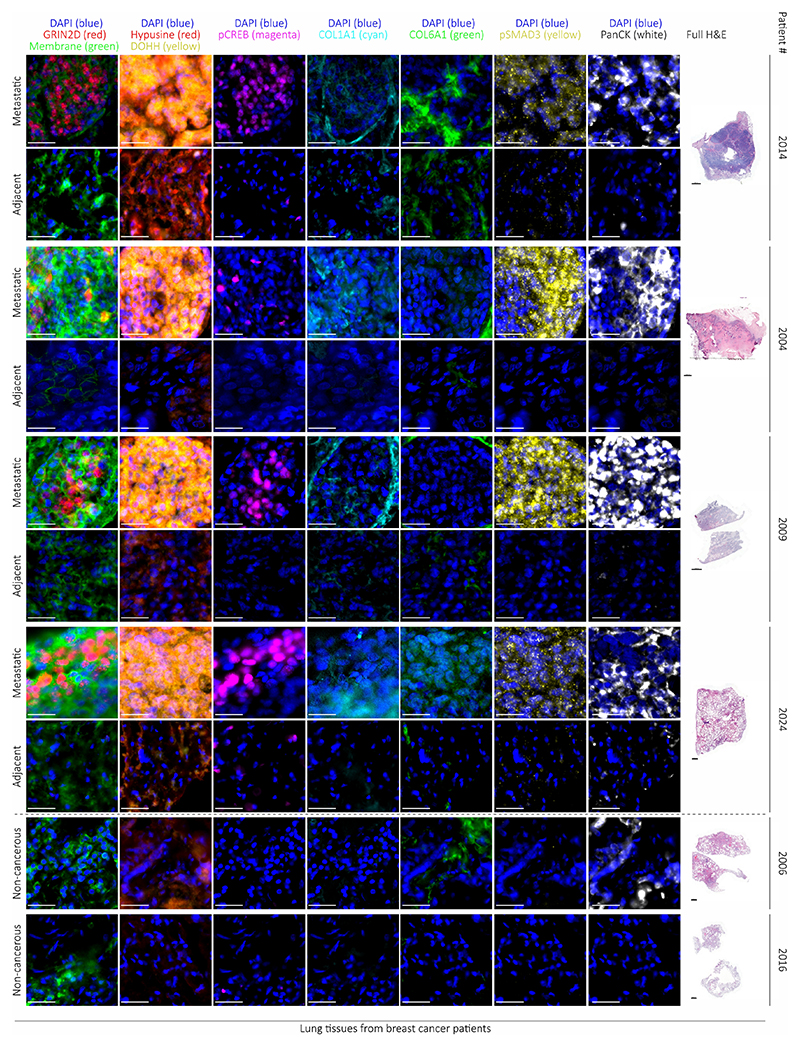
Representative images of IHC performed in breast cancer patients Left: GRIN2D, Hypusine, DOHH, phosphorylated CREB, COL1A1, COL6A1, phosphorylated SMAD3 and PanCK detected in metastatic and adjacent lungs from the UPTIDER breast cancer patients (*n* = 7), assessed by multiplex immunohistochemistry. Blue = DAPI nuclear staining, green = membrane marker ATP1A1; red = Hypusine, yellow = DOHH; magenta = phosphorylated CREB; cyan = COL1A1; green = COL6A1; yellow = phosphorylated SMAD3; white = PanCK. Scale bars = 50 μm. Membrane marker ATP1A1 has been added to identify surface localization of GRIN2D. Quantification for all patients, *n* = 7 (for a total of 5 regions per patient) is shown on Extended Data Figure 5c. Right: Representative images of H&E stainings of the whole lungs for each patient, scale bars = 2 mm. H&E and multiplex immunohistochemistry stainings originate from non-consecutive cuts.

**Figure 5:**
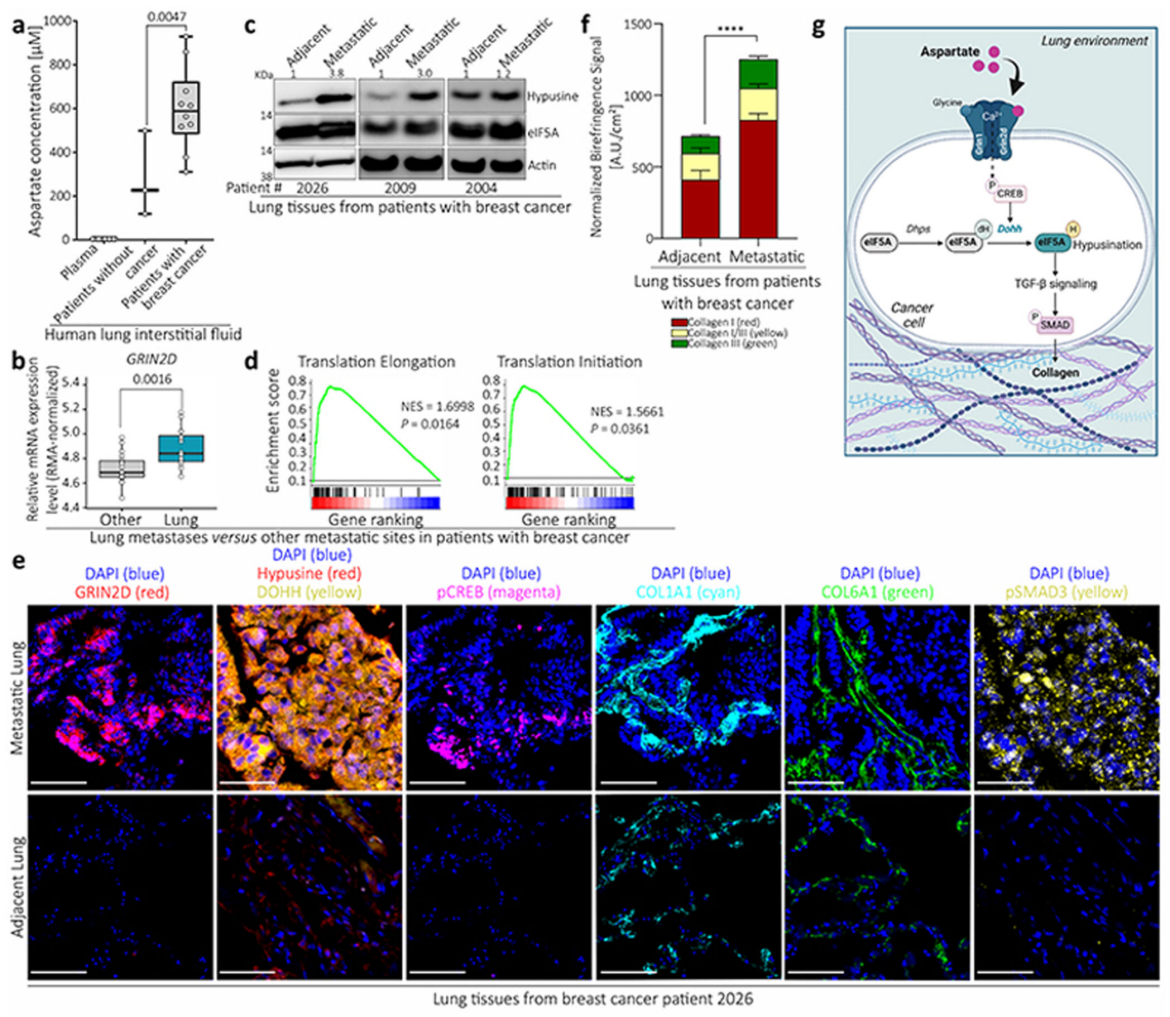
Evidence for aspartate signaling in lung metastases of breast cancer patients **a**. Aspartate concentration in plasma or lung interstitial fluid of breast cancer patients without detected lung metastases (*n* = 10) *vs* non-cancer patients (*n* = 3). One-way ANOVA (*P*<0.0001) with Tukey’s multiple-comparison tests, *P*=0.0121 plasma *vs* lung interstitial fluid of non-cancer patients and *P*<0.0001 plasma *vs* lung interstitial fluid of breast cancer patients. **b**. *GRIN2D* expression in lung *vs* bone/brain/liver metastases from breast cancer patient biopsies. *N* = 16 lung metastases and *n* = 20 non-lung metastases. *P*-value based on differential expression analysis with *limma*. **c**. Hypusine detected in lung metastases and adjacent non-cancerous tissues from breast cancer patients of the UPTIDER program. Hypusine quantification relative to total eIF5A shown on top of each lane. **d**. GSEA enrichment for translation elongation (left) and initiation (right) gene sets based on differential expression analysis comparing lung *vs* non-lung (bone/brain/liver) metastases of breast cancer patients. *P*-values based on *fgsea*’s adaptive multilevel splitting Monte Carlo approach. **e**. Representative image of GRIN2D, Hypusine, DOHH, phosphorylated CREB, COL1A1, COL6A1 and phosphorylated SMAD3 detected in metastases and adjacent non-cancerous lung tissues of breast cancer patients of the UPTIDER program (*n* = 7 patients). Blue=DAPI nuclear staining, red=GRIN2D (scale bars=100μm); red=Hypusine, yellow=DOHH (40μm); magenta=pCREB (100μm); cyan=COL1A1 (100μm); green=COL6A1 (100μm); yellow=pSMAD3 (40μm). Representative images for additional patients are shown in Extended Data Figure 6. **f**. Quantification of linearized collagen detected using Picrosirius Red in metastases and adjacent non-cancerous lung tissues of breast cancer patients of the UPTIDER program (*n* = 5). Significant collagen red increase (*P*<0.0001). Data normalized by metastasis area (cm^2^), shown as mean ± SEM. Two-way ANOVA with Šídák’s multiple-comparison tests. Representative image shown in Extended Data Figure 5d. **g**. Schematics of the identified mechanism: P=Phosphorylation; dH=deoxyhypusine; H=hypusine. For **a,b** box hinges indicate 1^st^/3^rd^ quartiles, mid-lines represent medians, and whiskers span the data range. Individual data points indicated by dots.

Finally, we investigated lung metastases from patients with breast cancer collected via the rapid *postmortem* tissue donation program UPTIDER (KU/UZ Leuven)^18^ and publicly available gene expression data. In line with our mouse data, we found that breast cancer patients had increased aspartate concentrations in their lung interstitial fluid (*n*=3 patients without cancer, *n*=10 patients with breast cancer, Figure 5a). Accordingly, lung metastases (*n*=16) showed increased expression of *GRIN2D* (but not other NMDA receptor subunits) compared to other metastatic sites (bone, brain and liver, *n*=20 jointly) in a cohort of metastatic breast cancer patients (Figure 5b, Extended Data Figure 5a). Lung metastases also displayed higher eIF5A hypusination compared to adjacent (non-tumor) tissues from patients with breast cancer (*n*=3, Figure 5c). Moreover, we also identified an increased translation initiation/elongation signature in lung metastases (*n*=16) of breast cancer patients compared to metastases from other organs (*n*=20) (Figure 5d). Additionally, we found that lung metastases showed increased GRIN2D, hypusine, DOHH, phosphorylated CREB, phosphorylated SMAD3, COL1A1, COL6A1 and collagen I-III compared to adjacent (non-tumor) tissues in breast cancer patients (*n*=7, Figure 5e,f, Extended Data Figures 5b-d,6). Thus, we provide evidence for aspartate signaling in lung metastases from patients with breast cancer.

Taken together, we discovered a novel function of aspartate as signaling molecule in cancer cells promoting metastasis aggressiveness. Specifically, we found that pulmonary aspartate activates the NMDA receptor which promotes eIF5A hypusination and in turn an alternative translation program driving collagen synthesis (Figure 5g). Aspartate is very low in blood plasma, and was considered to be predominantly important for fueling protein and nucleotide synthesis as well as maintaining redox homeostasis^19^. Several cancers have been shown to express the NMDA receptor^20,21^. However, its role in cancer cells has been mainly linked to pseudosynaptic behavior and glutamate metabolism^21–23^. In addition, it was shown that TGF-β can induce eIF5A hypusination in high-grade metastatic breast cancers, increasing the translation of the pro-metastatic non-receptor tyrosine kinase PEAK1^24^. Here, we add a novel mechanism by identifying extracellular aspartate as a signaling molecule binding the NMDA receptor, NMDA receptor activity as an inducer of eIF5A activity via hypusination and eIF5A-induced translation as a regulator of collagen synthesis.

Cancer cell plasticity is essential for metastasis formation^25,26^, and this plasticity often depends on translational reprograming^27^. Here, we show for the first time that nutrient priming of the lung by the secretome of primary breast tumors leads in disseminated cancer cells to the induction of an alternative translational program resulting in metastasis aggressiveness. Our findings may open new research lines on aspartate as an *extracranial* signaling molecule regulating alternative translation and nutrient priming of the pre-metastatic niche as an inducer of metastases aggressiveness. Moreover, the existence of clinically approved NMDA receptor and DOHH inhibitors may facilitate the translation of these findings towards clinic impact.

## Methods

### Cell culture

Human MCF10A breast epithelial cells, HEK293T epithelial cells, mouse 4T1 mammary gland cancer cells and human HUH7 hepatocellular carcinoma cells were obtained from ATCC. Mouse EMT6.5 mammary gland cancer cells were provided by R. Anderson (Peter MacCallum Cancer Center). Mouse B16F10 melanoma cells were provided by Prof. Ilaria Elia (KU Leuven). Mouse 4T07 cells were provided by Prof. Ana Gomes (H Lee Moffitt Cancer Center). MCF10A cells expressing H-RasV12 (MCF10A H-Ras^12^) were generated as previously described^9^. Human MCF10A H-Ras^v12^ cells were maintained in Dulbecco’s Modified Eagle’s Medium F12 (DMEM/F12, Life Technologies) supplemented with 5% heat-inactivated horse serum (Life Technologies), 1% penicillin (50 U ml^-1^, Life Technologies), 1% streptomycin (50 μg ml^-1^, Life Technologies), 0.5 μg ml^-1^ hydrocortisone, 100 ng ml^-1^ cholera toxin, 10 μg ml^-1^ human insulin and 20 ng ml^-1^ recombinant human Epidermal Growth Factor (EGF). Human HEK293T and HUH7 cells were cultured in high-glucose (4.5 g l^-1^) Dulbecco’s modified Eagle’s medium (DMEM, Life Technologies) supplemented with 10% heat-inactivated fetal bovine serum (Life Technologies), 1% penicillin (50 U ml^-1^, Life Technologies) and 1% streptomycin (50 μg ml^-1^, Life Technologies). Mouse 4T1, 4T07 and EMT6.5 cells were cultured in Roswell Park Memorial Institute (RPMI, Life Technologies) 1640 medium supplemented with 10% heat-inactivated fetal bovine serum (Life Technologies), 1% penicillin (50 U ml^-1^, Life Technologies) and 1% streptomycin (50 μg ml^-1^, Life Technologies). Mouse B16F10 cells were cultured in Roswell Park Memorial Institute (RPMI, Life Technologies) 1640 medium supplemented with 1% sodium pyruvate (1 mM, Gibco), 1% HEPES (10 mM), 10% heat-inactivated fetal bovine serum (Life Technologies), 1% penicillin (50 U ml^-1^, Life Technologies) and 1% streptomycin (50 μg ml^-1^, Life Technologies). All cells were screened to be mycoplasma free via routine testing with the MycoAlert Mycoplasma Detection Kit (Lonza) and the MycoStrip Mycoplasma Detection Kit (InvivoGen). For 3D spheroid growth assays, cells were plated on top of a soft agar mixture^9,28^ of 50% agar and 50% Lung-Like Medium (LLM, Supplementary Table 1). 4T1 and MCF10A H-Ras^v12^ cells were cultured for 3 to 5 days in freshly made (less than 3 days) LLM supplemented with or without 700 μM aspartate and with 5% heat-inactivated horse serum (Life Technologies) 1% penicillin (50 U ml^-1^, Life Technologies) and 1% streptomycin (50 μg ml^-1^, Life Technologies). MCF10A H-Ras^v12^ cells were further supplemented with 0.5 μg ml^-1^ hydrocortisone, 100 ng ml^-1^ cholera toxin, 10 μg ml^-1^ human insulin and 20 ng ml^-1^ recombinant human epithelial growth factor (EGF). 1.5% of reduced growth factor Matrigel (Corning) without phenol red and 1.5% Collagen I, Rat Tail (45 μg ml^-1^, Gibco) were added into LLM before seeding by using tips precooled to -20°C. The NMDA receptor inhibitor MK-801 (Tocris, 0924) was used at a final concentration of 50 μM. The CREB inhibitor Compound-3i, 666-15 (Selleck Chem, S8846) was used at a final concentration of 600 nM. The TGF-β inhibitor LY364947 (Selleck Chem, S2805) was used at a final concentration of 5 μM. Tunicamycin (Merck Millipore, T7765) was used at 0.1 μg ml^-1^. Representative images of 3D spheroids were taken at the end of the experiment (3-5 days) with the Motic Images Plus 2.0 software (Motic) and were quantified using a custom script in ImageJ.

### Generation of knockdown and overexpression cell lines

**Extended Data Figure 7:**
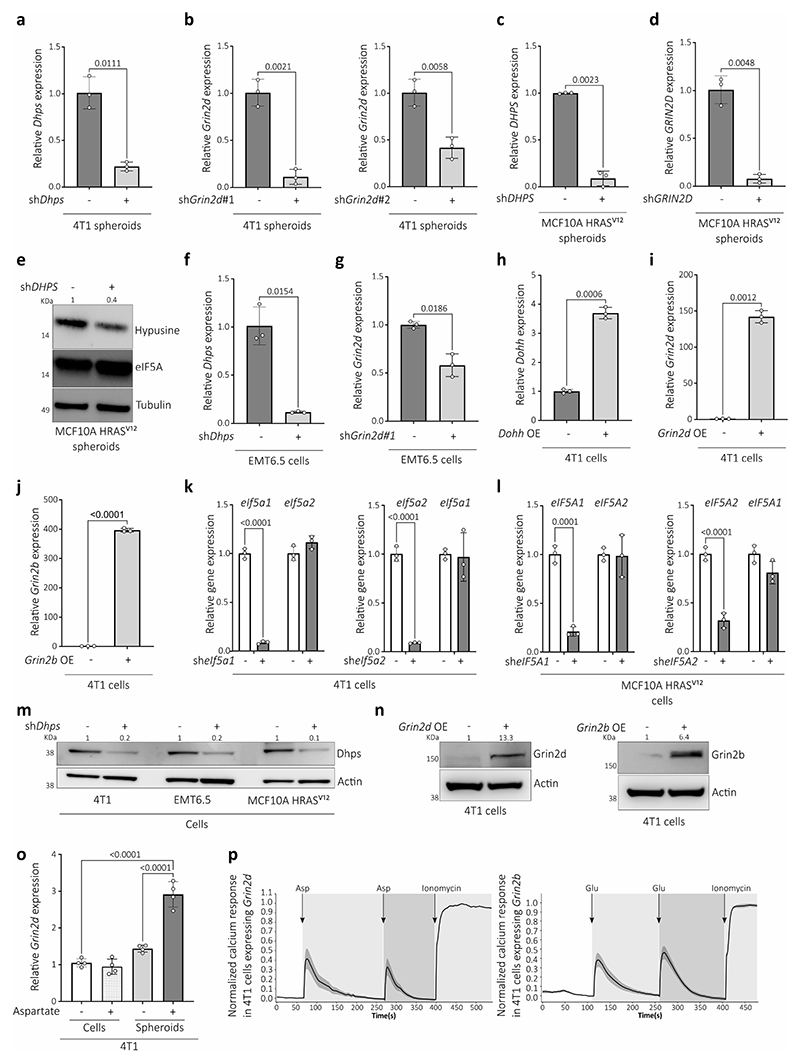
Protein and mRNA expression of genetically modified breast cancer cells and method validation **a**. Relative mRNA expression levels of *Dhps* in 4T1 spheroids silenced for *Dhps* or scramble shRNA. Data are normalized relative to the average of the control (shRNA against scramble sequence) condition. Bars represent averages, and single dots individual replicates. Error bars represent ± s.d (*n* = 3 independent replicates). Unpaired two-tailed *t*-test with Welch correction. **b**. Relative mRNA expression levels of *Grin2d* in 4T1 spheroids silenced for *Grin2d* or scramble shRNA. Data are normalized relative to the average of the control (shRNA against scramble sequence) condition. Bars represent averages, and single dots individual replicates (*n* = 3 independent replicates). Unpaired two-tailed *t*-test with Welch correction. **c**. Relative mRNA expression levels of *DHPS* in MCF10A H-Ras^v12^ spheroids silenced for *DHPS* or scramble shRNA. Data are normalized relative to the average of the control (shRNA against scramble sequence) condition. Bars represent averages, and single dots individual replicates (*n* = 3 independent replicates). Unpaired two-tailed *t*-test with Welch correction. **d**. Relative mRNA expression levels of *GRIN2D* in MCF10A H-Ras^v12^ spheroids silenced for *GRIN2D* or scramble shRNA. Data are normalized relative to the average of the control (shRNA against scramble sequence) condition. Bars represent averages, and single dots individual replicates (*n* = 3 independent replicates). Unpaired two-tailed *t*-test with Welch correction. **e**. Hypusine levels in MCF10A H-Ras^v12^ cells silenced for *DHPS* or scramble shRNA. Quantification of hypusine signal normalized over total eIF5A signal is indicated on top of each lane. **f**. Relative mRNA expression levels of *Dhps* in EMT6.5 cells silenced for *Dhps* or scramble shRNA. Data are normalized relative to the average of the control (shRNA against scramble sequence) condition. Bars represent averages, and single dots individual replicates (*n* = 3 independent replicates). Unpaired two-tailed *t*-test with Welch correction. **g**. Relative mRNA expression levels of *Grin2d* in EMT6.5 cells silenced for *Grin2d* or scramble shRNA. Data are normalized relative to the average of the control (shRNA against scramble sequence) condition. Bars represent averages, and single dots individual replicates (*n* = 3 independent replicates). Unpaired two-tailed *t*-test with Welch correction. **h**. Relative mRNA expression levels of *Dohh* in 4T1 cells expressing an overexpression vector for *Dohh* or an empty vector. Data are normalized relative to the average of the control (empty vector) condition. Bars represent averages, and single dots individual replicates (*n* = 3 independent replicates). OE = overexpression. Unpaired two-tailed *t*-test with Welch correction. **i**. Relative mRNA expression levels of *Grin2d* in 4T1 cells expressing an overexpression vector for *Grin2d* or an empty vector. Data are normalized relative to the average of the control (empty vector) condition. Bars represent averages, and single dots individual replicates (*n* = 3 independent replicates). Unpaired two-tailed *t*-test with Welch correction. **j**. Relative mRNA expression levels of *Grin2b* in 4T1 cells expressing an overexpression vector for *Grin2b* or an empty vector. Data are normalized relative to the average of the control (empty vector) condition. Bars represent averages, and single dots individual replicates (*n* = 3 independent replicates). Unpaired two-tailed *t*-test with Welch correction. **k**. Relative mRNA expression levels of *eIf5a1* and *eIf5a2* in 4T1 cells silenced for *eIf5a1 or eIf5a2* or scramble shRNA. Data are normalized relative to the average of the control (shRNA against scramble sequence) condition. Bars represent averages, and single dots individual replicates. Error bars represent ± s.d (*n* = 3 independent replicates). Two-way ANOVA with Šídák’s multiple-comparison tests. **l**. Relative mRNA expression levels of *eIF5A1* and *eIF5A2* in MCF10A H-Ras^v12^ cells silenced for *eIF5A1 or eIF5A2* or scramble shRNA. Data are normalized relative to the average of the control (shRNA against scramble sequence) condition. Bars represent averages, and single dots individual replicates. Error bars represent ± s.d (*n* = 3 independent replicates). Two-way ANOVA with Šídák’s multiple-comparison tests. **m**. Dhps levels in 4T1, EMT6.5 and MCF10A H-Ras^v12^ cells silenced for *DHPS* or scramble shRNA. Quantification of Dhps signal normalized over actin signal is indicated on top of each lane. **n**. Left: Grin2d levels in 4T1 cells expressing an overexpression vector for *Grin2d* or an empty vector. Quantification of Grin2d signal normalized over actin signal is indicated on top of each lane. Right: Grin2b detected in 4T1 cells expressing an overexpression vector for *Grin2b* or an empty vector. Quantification of Grin2b signal normalized over actin signal is indicated on top of each lane. **o**. Relative mRNA expression levels of *Grin2d* in 4T1 cells or spheroids cultured as a 2D monolayer in RPMI (left) or as 3D spheroids in lung-like medium (right) with or without aspartate. Data are normalized relative to the average of the control (2D no aspartate) condition. Bars represent averages, and single dots individual replicates (*n* = 4 independent replicates). One-way ANOVA (*P* < 0.0001) with Tukey’s multiple-comparison tests. **p**. Average ionomycin-normalized calcium response traces in 4T1 cells overexpressing *Grin2d* (left) or *Grin2b* (right). The shaded ribbons represent standard errors of the mean at each time point in each sample, while the grey rectangles indicate sequential additions of aspartate (left) or glutamate (right) plus ionomycin at the times indicated by the arrow. A representative experiment (*n* = 14 cells for *Grin2d* OE and *n* = 15 cells *Grin2b* OE) out of 2 independent experiments is shown.

*Dhps, Grin2d, eIf5a1* and *eIf5a2* silenced cell lines were generated using the lentiviral pLKO-shRNA1.5 vector expressing shRNAs against *Dhps, Grind2d, eIf5a1* or *eIf5a2*. The abovementioned pLKO plasmids were obtained from the Belgian Coordinated Collections of Microorganisms (BCCM). The pLKO plasmid (SHC016) expressing a non-targeting shRNA sequence was used as control and was obtained from the BCCM. Briefly, lentiviral particles were produced in HEK293T cells. Transduction of 4T1, EMT6.5 and MCF10A H-Ras^v12^ was performed overnight and the medium was replaced the next day. Polyclonal cells were selected with puromycin (2 μg ml^-1^ for 4T1 cells, 1 μg ml^-1^ for EMT6.5 cells and 0.3 μg ml^-1^ for MCF10A H-Ras^v12^). To generate the *Dohh, Grin2d* and *Grin2b* overexpressing cell line, the pLVX-IRES-Hyg plasmid (Takara Bio, #632182) was digested with BamHI and XhoI (NEB). A codon optimized coding sequence of *Dohh, Grin2d* or *Grin2b* was purchased as a gBlock (Integrated DNA Technologies, IDT) and inserted into the pLVX-IRES-Hyg backbone using Gibson assembly (NEB). The empty pLVX-IRES-Hyg plasmid was used as control. Lentiviral particles were produced in HEK293T cells and cells were transduced overnight and fresh medium was replaced the next day. Polyclonal cells were selected with hygromycin at 100 μg ml^-1^. A list of sequences used to generate knockdown and overexpression cell lines is described in Supplementary Table 2. Validation of knockdown and overexpression cell lines by means of quantitative PCR and western blot analysis is present in Extended Data Figure 7.

### *In vivo* mouse experiments

All animal experiments were approved by the Institutional Animal Care and Research Advisory Committee of KU Leuven (ECD nos. P025/2020) in compliance with all relevant ethical regulations. Mice were housed under a regimen of 12 h light-12 h dark in non-SPF (conventional) facility with a constant supply of food and water. Temperature was checked daily and maintained at 22 ±2 °C, humidity was checked daily and maintained between 45-70%. Sample size was determined using power calculations with *B* = 0.8 and *P* < 0.05 based on preliminary data and in compliance with the 3R system: replacement, reduction, refinement. All mice were randomized before injections and samples were analyzed in a blinded manner. Female BALB/C (Envigo) mice (aged 6-8 weeks) were injected with 4T1 or EMT6.5 cells in the mammary fat pad (m.f.p., 1x10^6^ cells) in 50 μl PBS or intra venously (i.v., 25x10^3^ cells or 1x10^5^) in 100 μl PBS or intrasplenic (i.s., 25x10^3^ cells; spleen was surgically removed two minutes after injection: mice were anesthetized with 2% isofluorane in pure oxygen at 2L min^-1^ and Carprofen at 5mg kg^-1^ was administered subcutaneously before and after surgery) in 50 μL PBS. Mice were euthanized after 21-23 days (m.f.p.), 13-17 days (i.v.) or 14 days (i.s.) by intraperitoneal (i.p.) injection of 10 μl g^-1^ containing ketamine (100 mg kg^-1^)-xylazine (10 mg kg^-1^) for experiments involving organ dissociation or 50 μl of a 60 mg ml^-1^ Dolethal (pentobarbital sodium) solution (Vetoquinol) for experiments involving metastasis picking or IHC stainings. Primary tumor volumes (calculated using the following formula: *V* = (π/6) × length × width × height) were measured during every experiment using a manual calliper and weighted at the end of the experiment. During the course of the experiments, mice were monitored for detection of humane end points, determined using a score sheet (tumor size of 1.8 cm^3^, loss of ability to ambulate, labored respiration, surgical infection or weight loss over 10% of the initial body weight). For all experiments the tumor volume did not exceeded 1.8 cm^3^.

### Generation of Tumor-Secreted Factors and *in vivo* (pre-)metastatic niche formation

The induction of pre-metastatic niche formation was performed as previously described^6^. Briefly, for collecting Tumor Secreted Factors (TSF), 4T1 or EMT6.5 derived primary tumors were resected after 17 days from m.f.p. injection, cut into smaller pieces and incubated at 37 °C in 15 ml g^-1^ of tumor in Dulbecco’s Modified Eagle’s Medium (DMEM, Life Technologies) supplemented with 1% penicillin (50 U ml^-1^, Life Technologies) and 1% streptomycin (50 μg ml^-1^, Life Technologies). For control medium formation, the same medium was incubated in parallel. After 72 h, medium was filtered with a 70 μm cell strainer and centrifuged at 1,000 x g for 10 minutes. Supernatants from three biologically independent tumors were pooled together to cover for biological variability and 20 mM HEPES was added, subsequently medium was filtered with a 0.2 μm filter and stored at 4 °C for maximum 5 days from collection. Female BALB/C mice (6 weeks old) were then injected i.v. with 200 μl of TSF or control medium three times per week for three weeks and subsequently either euthanized (pre-metastatic niche: for lung interstitial fluid extraction or single-cell RNA sequencing), or injected i.v. with 25x10^3^ CD90.1-expressing 4T1 or EMT6.5 cancer cells and then euthanized after 16 (4T1) or 14 (EMT6.5) days after cancer-cell injection. Subsequently, lungs were analyzed for single-cell RNA sequencing, flow cytometry or protein analysis from snap-frozen metastatic tissues.

### *In vivo* aspartate priming of the lung microenvironment

Female BALB/C mice (6 weeks old) were injected with daily intra peritoneal (i.p.) injections of 20 mM aspartate dissolved in 100 μl PBS (pH 7.4), or PBS (pH 7.4) for 10 days. Afterwards, mice were either euthanized with 50 μl of a 60 mg ml^-1^ Dolethal (pentobarbital sodium) for lung interstitial fluid collection at 0, 4, 7, 10 or 14 days after the last i.p. injection or mice were injected with cancer cells for metastatic burden evaluation. Specifically, 4T1 or EMT6.5 expressing CD90.1 were injected i.v. (1x10^5^ cells) in 100 μl of PBS and after 14 (4T1) or 13 (EMT6.5) days, lungs were dissociated for flow cytometry analysis or metastatic tissues were snap-froze for protein analysis.

### *In vivo* Memantine treatment

Female BALB/C mice (6 weeks old) were injected (m.f.p.) with 4T1 cells expressing CD90.1, after 5 days (when the tumor nodule was palpable) mice were treated with daily i.p. injections (100 μL) of Memantine-HCl (5 mg kg^-1^, 1 mg ml^-1^) or vehicle (PBS) for 19 days. Afterwards, mice were euthanized for metastatic burden evaluation by flow cytometry analysis.

### RNA isolation, qPCR and Droplet Digital PCR gene expression analysis

Total RNA from cell lines was isolated using the TRIzol Reagent (Thermo Fisher Scientific) and the isolated RNA was quantified using the NanoDrop One Microvolume UV-Vias spectrophotometer (Thermo Fisher Scientific). RNA was reverse transcribed using the qScript cDNA Synthesis kit (Quantabio) and was measured by quantitative PCR (qPCR) using SYBR Green PCR Master Mix, Low ROX (Quantabio) on the 7500 Fast Real Time PCR System (Applied Biosystems, Life Technologies). Relative mRNA expression levels were determined by normalization relative to an endogenous housekeeping gene (Cyclophillin B or Rpl19). Droplet digital PCR was performed to determine the number of copies for *Grin2b* and *Grin2d* in 4T1 lung metastases (m.f.p., 25 days) using the QX200 Droplet Digital PCR system. Specifically, lung metastases were picked and ground, and total RNA from frozen tissues was isolated using TRIzol™ Reagent (Thermo Scientific). cDNA synthesis was performed using the qScript cDNA synthesis kit (Quantabio). Droplet digital PCR was performed following Bio-Rad guidelines (Bio-Rad). Specifically, a solution containing 21 uL of 2X ddPCR Supermix for probes (without dUTPs), 20x FAM Taqman probe (for *Grin2b* expression), 20x Hex Taqman probe (for *Grin2d* expression), 7 μL of diluted cDNA (for a final concentration of 1750 ng) and 1.4 μL of nuclease-free water. Droplets were created using a QX200 Droplet Generator and transferred to a 96-well PCR plate heat-sealed using foil sheets (Pierceable Foil Heat Seal, Bio-Rad, 1814040) and the PX1 PCR plate sealer. The Bio-Rad T100 thermal cycler system was used to amplify the droplets, starting with 3 min enzyme activation at 95 °C, followed by 40 cycles of 95 °C, 30 sec and 58 °C, 30 sec, 72 °C for 1 min, followed by 72 °C for 5 min. Fluorescence of each reaction was measured using the QX200 Droplet Reader (Bio-Rad) and the results were analyzed using the QuantaSoft software v.1.7.4.0917 (Bio-Rad) subsequent to threshold setting on positive controls. The resulting number of positive and negative droplets were used to calculate the concentration of cDNA copies μL^-1^ of the target genes in the final reaction. A list of primers is provided in Supplementary Table 3.

### Protein extraction and western blot analysis

Cultured 3D spheroids were collected in dPBS and lysed in RIPA buffer (Thermo Fisher Scientific, 89901) supplemented with protease (Merck Sigma, 5892970001) and phosphatase (Merck Sigma, 4906845001) inhibitors. Frozen tissues were ground with a tissue lyser and then incubated with RIPA buffer. Protein lysates were quantified with the Pierce BCA Protein Assay Kit (Thermo Fisher Scientific, 23225) and 10-20 μg of proteins were loaded on a precast gel NuPAGE Novex 4-12% Bis-Tris (Thermo Fisher Scientific, NP0336BOX). Proteins were subsequently transferred to a nitrocellulose membrane using the iBlot2 dry transferring system (Thermo Fisher Scientific, IB301031). Transferred membranes were blocked for 1 h at room temperature in a blocking solution of 5% milk or 5% bovine serum albumin in Tris Buffer Saline 005% Tween-20 (TBS-T). Subsequently, membranes were incubated overnight at 4 °C with primary antibodies against hypusine (EMD Millipore, ABS1064), eIF5A (BD Biosciences, 611976), phosphorylated CREB (Ser133) (Cell Signaling Technologies, 9198S), CREB (Cell Signaling Technologies, 9104S), phosphorylated SMAD3 (S423+S425) (Abcam, EP823Y), SMAD3 (Cell Signaling Technologies, 9513S), Grin2d (Novus Biological, NBP2-94573), Grin2b (Abcam, Ab93610), Dhps (Santa Cruz Biotechnology, sc-365077), puromycin (Merck Sigma, MABE343), phosphorylated eIF2α (Cell Signaling Technologies, 9721S), ATF4 (Cell Signaling Technologies, 11815S), β-Actin (Merck Sigma, A5441), β-Tubulin (Cell Signaling Technologies, 2146S). The primary antibodies were used at 1:1,000 dilution in 5% bovine serum albumin in TBS-T, except for anti-Hypusine (1:2,000), anti-ATF4, anti-Grin2d and anti-Dhps (1:500) and anti-β-Actin (1:10,000) antibodies. The day after, membranes were incubated with HRP-linked anti-rabbit (Cell Signaling Technologies, 7074S) or anti-mouse (Cell Signaling Technologies, 7076S) secondary antibodies used at 1:5,000 dilution in 5% milk in TBS-T. Bound antibodies were imaged using an ImageQuant LAS 4000 (GE Healthcare). For gel source data, see Supplementary Figure 1.

### SUnSET assay

Puromycin incorporation into newly synthesized protein was used to evaluate overall protein synthesis as previously described^29^. 4T1 and MCF10A H-Ras^v12^ 3D spheroids were cultured for 5 days in LLM with or without aspartate supplementation, afterwards spheroids were starved for 1 hr in Hank’s Balanced Salt Solution (HBSS, Gibco 14025-050) and then reactivated in LLM with or without aspartate and incubated with the protein synthesis inhibitor Cycloheximide (100 nM, Merck Sigma, C7698) or 0.001% DMSO for 1.5 hr; puromycin was added during the last 20 minutes of incubation at 10 μg mL^-1^. Next, colonies were collected for protein extraction, loaded on a precast gel and transferred as described above. Before blocking, membranes were blotted with Ponceau red and puromycin incorporation was evaluated via western blotting as described above.

### Polysome profiling and sequencing analysis

In order to determine genes whose protein translation is upregulated as a result of aspartate-driven NMDA-receptor signaling and downstream eIF5A hypusination, control (shSCR), GRIN2D-knockdown (shGRIN2D), and DHPS-knockdown (shDHPS) 4T1 spheroids were cultured for 5 days in LLM supplemented with 700 μM aspartate (ASP) and, in the case of control spheroids, also under no aspartate supplementation (NoASP-shSCR). Polysome isolation was performed as previously described^30,31^. Briefly, spheroids were collected and washed in ice-cold cycloheximide-supplemented PBS, lysed in hypotonic buffer (2.5mM MgCl_2_, 5mM TRIS pH 7.5, 1.5mM KCl supplemented with RNAse and protease inhibitors) and permeabilized by sequential addition of 0.5% Triton X-100 and 0.5% SDS. Lysates were then centrifuged for 7 minutes at 16,000 x g. For each condition, supernatant containing the cytosolic lysate was then loaded on top of a non-linear sucrose gradient (5% - 34% - 55%) and further ultracentrifuged at 200,000 x g for 2 hours at 4 °C. Total RNA input (~10% of the cytosolic lysate) was immediately lysed in Trizol solution (TriPure Isolation Reagent, ref. 11667165001, Sigma), snap-frozen in liquid nitrogen, and kept for RNA extraction (see section Total RNA sequencing). Pplysome fractions were collected using a piston gradient fractionator (BioComp). Matched polysomal (efficiently translated mRNA; associated with >3 ribosomes) and sub-polysomal (associated with <3 ribosomes) gradient fractions from 4 independent spheroid pellets per condition were then lysed in Trizol, snap-frozen in liquid nitrogen and kept at -80 °C for RNA extraction. mRNA library preparation was performed using the Illumina Stranded mRNA Prep kit (ref. 20040534, Illumina). After quantification with qPCR, libraries were sequenced on a NovaSeq 6000 System (Illumina) using the NovaSeq 6000 S4 Reagent kit v1.5 (ref. 20028312, Illumina), generating 2x150bp paired-end reads. The resulting reads were trimmed for adaptors and low-quality base calls using *Trim Galore!* (v0.6.6; https://github.com/FelixKrueger/TrimGalore), after which quality control was performed with *FastQC* (v0.11.9; https://www.bioinformatics.babraham.ac.uk/projects/fastqc). High-quality reads were then mapped to the mm39 reference mouse genome (GRCm39) using STAR (v2.6.1; https://github.com/alexdobin/STAR) and quantified using *Salmon* (v1.4.0; https://combine-lab.github.io/salmon). Gene counts for all 4 matched polysomal/sub-polysomal sample pairs collected under all 4 conditions of interest (ASP shSCR, ASP shGRIN2D, ASP shDHPS, NoASP shSCR) were processed simultaneously within the *DESeq2* (v1.34.0; https://bioconductor.org/packages/release/bioc/html/DESeq2.html) framework^32^. In brief, *Salmon*-derived counts were first rounded to the closest integers, after which genes with no expression in at least 3 out of 4 replicates for at least one of the 8 condition/fraction combinations were excluded from the analysis. Integer counts for the remaining 17757 genes were then modelled within *DESeq2*, using a design accounting for the paired nature of the polysomal/sub-polysomal fractions for each replicate and condition, of the form

Counts~Condition+Condition:Replicate+Condition:Fraction

where : denotes an interaction, *Condition* denotes either of ASP shSCR, ASP shGRIN2D, ASP shDHPS, or NoASP shSCR, *Fraction* denotes either of the sub-polysomal (reference level) or polysomal fractions, and *Replicate* = 1 ⋯ 4. Based on this design, the coefficients for the interaction terms *Condition: Fraction* directly represent the average log fold changes (log_2_ FC) in mRNA levels between the polysomal/sub-polysomal fractions for each of the 4 conditions of interest, accounting for replicate pairing. The differences between these log fold changes for the ASP shSCR condition and those for all other 3 conditions thus provide a measure of whether protein translation for a specific gene is altered in the presence of aspartate (ASP shSCR *vs* NoASP shSCR) or due to either NMDA-receptor activity (ASP shSCR *vs* ASP shGRIN2D) or hypusine production (ASP shSCR *vs* ASP shDHPS). These log fold-change differences were determined directly within *DESeq2*, further being subject to shrinkage using the *ashr* adaptive shrinkage estimator^33^ (https://github.com/stephens999/ashr). The resulting shrunken log fold-change differences (Δlog_2_ FC) and their associated p-values were then combined to derive a translation ranking metric TRM = − Δlog_2_ FC × log_10_(p-value), whereby highly positive/negative values of TRM indicate genes for which protein translation is highly up/downregulated, respectively.

Pre-ranked gene-set enrichment analysis (GSEA^34^; https://www.gsea-msigdb.org/gsea) was then performed, using the *R* package *fgsea*^35^ (v1.20.0; https://github.com/ctlab/fgsea; multilevel implementation with 10000 initial permutations and no lower bound for p-value estimation), for each of the 3 comparisons of interest, based on the ranking metric TRM, and considering a collection of 3065 mouse gene sets. The latter comprised all HALLMARK and CURATED (C2) gene sets in the Broad Institute Molecular Signatures Database (MSigDB^36,37^; https://www.gsea-msigdb.org/gsea/msigdb) except for those in category C2:CGP, all of them obtained via the *R* package *msigdbr* (v7.4.1; https://igordot.github.io/msigdbr), plus all KEGG^38^ (https://www.genome.jp/kegg) metabolism gene sets, obtained by querying KEGG’s REST API (https://www.kegg.jp/kegg/rest). Only gene sets containing between 3 and 1500 genes were considered in this analysis. Gene sets with positive normalized enrichment scores (NES) in all 3 comparisons were identified and ranked based on their average NES among all comparisons, with the top 15 of these being shown in the manuscript. To build the word cloud with the top enriched terms based on increased translation in ASP shSCR *vs* all other 3 conditions, combined, average shrunken log fold-change differences (Δlog_2_ FC) between the Asp shSCR condition and all other 3 conditions were first determined for each gene, again directly within *DESeq2*. The latter were then used, together with the associated p-values, to derive a global translation ranking metric, analogously as described above. This global translation ranking metric was then used to perform GSEA, identically as described above, after which upregulated gene sets were ranked based on their NES. Gene-set names were then split into individual words, or *terms*, and each of these terms were assigned a ranking percentile RP based on the ranking of their associated gene set, rounded up to multiples of 0.1%. A score for each individual term in each gene set was then computed as 100/RP (thus covering the range 1–1000), after which an overall score for each term was determined by averaging its scores among all gene sets in which the term appeared. The latter overall scores were then used as weights to create the word cloud, based on the 100 terms with the highest overall scores. Common and/or uninformative terms, such as prepositions (e.g. WITH, VIA, THROUGH…), conjunctions (e.g. AND, OR…), or generic terms (e.g. GENE, PATHWAY, REGULATION, TARGET…) were removed from the ranked list of terms prior to generating the word cloud.

### Total RNA sequencing

Total RNA fractions from ASP shSCR, ASP shGRIN2D, ASP shDHPS, and NoASP shSCR 4T1 spheroids, preserved from the input of the polysome profiling experiments, were subject to mRNA library preparation, sequencing, trimming, and mapping identically as described in the section Polysome profiling and sequencing analysis. Gene counts were again processed within the *DESeq2* framework, with *Salmon*-derived counts first rounded to the closest integers, and genes with no expression in at least 2 out of 4 replicates for at least one of the 4 conditions being excluded from the analysis. Integer counts for the remaining 19525 genes were then modelled within *DESeq2* to determine log fold changes between the ASP shSCR and NoASP shSCR conditions. The resulting (ASP shSCR *vs* NoASP shSCR) log fold changes (log_2_ FC) and their associated p-values for each gene were then combined to derive a ranking metric RM = − log_2_ FC × log_10_(p-value), whereby highly positive/negative values of RM indicate respectively genes with highly up/downregulated expression in the presence *vs* absence of aspartate. Pre-ranked GSEA was then performed based on this ranking metric RM, and otherwise identically as described in the section Polysome profiling and sequencing analysis. Gene sets were ranked based on their normalized enrichment score (NES), with the top 15 gene sets with highest positive NES being shown in the manuscript.

### Collagen staining

4T1 and MCF10A H-Ras^v12^ spheroids grown for 5 (4T1) or 4 days (MCF10A H-Ras^v12^) in LLM were transferred to 35-mm glass bottom dishes coated with fibronectin and allowed to loosely attach. Spheroids were subsequently fixed with 4% paraformaldehyde in PBS for 30 minutes, permeabilized with 0.5% Triton-X100 in PBS for 20 minutes and blocked for 30 minutes with 1% bovine serum albumin in PBS. Spheroids were then incubated overnight at 4 °C with primary antibody against Collagen I (Abcam, Ab34710; 1:500 dilution), washed with PBS and incubated for 1h with the Alexa Fluor 555-conjugated secondary antibody (Life Technologies, A31272). Next, spheroids were washed with PBS and nuclei were stained with 6 μM of 4’,6-diamidino-2-phenylindole (DAPI; Sigma Aldrich, D9542) for 15 minutes. Fluorescence Mounting Medium (Dako, S3023) was used to mount coverslips. Samples were imaged on a SP8X inverted confocal microscope (Leica Microsystem) equipped with a 405 nm and a white light laser. Z-stacks were performed using the LAS AF acquisition software (Leica Microsystem). Collagen-I fluorescence intensity quantification was performed with Imaris Image Analysis Software 9 (Bitplane) and normalized over total nuclei number identified with DAPI.

### Single-cell cytosolic Ca^2+^ live imaging and quantification

To evaluate cytosolic Ca^2+^ levels, 4T1 cells overexpressing *Grin2d* or *Grin2b* were plated in four-chamber 35-mm glass bottom dishes (Cellvis, Cat. D35C4-20-0-N) for 3 days. Notably, overexpression cells were used to account for comparability between isoform expression levels and to perform Ca^2+^ imaging requiring adherent conditions which results in low basal levels of *Grin2d* expression compared to aspartate stimulated spheroid growth (Extended Data Figure 7o). Cells were loaded for 45 minutes with 2 μM of the Ca^2+^ indicator Cal-520 AM (Aat Bioquest) diluted in cell culture medium with the addition of 0.02% of Pluronic™ F-127 (20% Solution in DMSO, Thermo Fischer Scientific) in a humidified incubator at 37°C and 5% CO_2_. The cells were washed once with cell culture medium, and the incubation was continued for 30 minutes to complete dye de-esterification. Immediately before the Ca^2+^ imaging experiments, the cell culture medium was replaced with a pre-warmed (37°C) modified Krebs solution (135 mM NaCl, 6.2 mM KCl, 1.2 mM MgCl_2_, 12 mM HEPES, pH 7.3, 11.5 mM glucose and 1.5 mM CaCl_2_). Glycine (required for NMDA receptor activation and always added as first treatment), glutamate and aspartate were resuspended into the modified Krebs solution and gently added (sequentially) to the chamber after 30 sec of basal acquisition to reach a final concentration of 921 μM for glycine, 774 μM for glutamate and 700 μM for aspartate. Before the end of each recording, cells were exposed to 5 μM ionomycin, a Ca^2+^ ionophore agent, to ensure adequate Cal520 loading and cell responses, and to determine the maximal Ca^2+^ responses. Imaging was performed using a Nikon eclipse Ti2 inverted fluorescence microscope (Nikon) equipped with excitation filter FF01-378/474/554/635 and dichroic mirror FF01-432/515/595/730 and emission filter 515/30, all from Semrock. Excitation was performed at 470 nm using a CoolLed pR-4000 (CoolLed). Emission was measured at 515 nm using an emission filter 515/30. Acquisition of the emitted fluorescent signal was performed using a pco.edge 4.2bi sCMOS camera (pCO) as a function of time (a frame per second). FIJI software was utilized to perform image analysis and to obtain background-corrected fluorescence intensities. FIJI software was utilized to perform image analysis and to obtain dynamic traces of background-corrected fluorescence intensity *vs* time F_C_(t) for each individual cell C measured in each sample (C = 1 … N, where N is the total number of cells identified in that sample). The background-corrected cytosolic calcium levels F_C_(t) for the different cells in each sample were first normalized relative to baseline levels as Δ(F/F_0_)_C_(t) = F_C_(t)/FC_0_ − 1. Here, FC_0_ is the baseline level for each cell C, determined as the median of F_C_(t) across 20 consecutive time points, common to all cells in a given sample, and defined within the time range (a) prior to the addition of the treatments of interest (in our case, glutamate and aspartate) and (b) displaying a high signal stability across all cells in the sample. Normalized calcium response amplitudes for the different added treatments T (where T = glycine, glutamate, aspartate, or ionomycin) were then determined for each cell C as Δ(F/F_0_)_C,T_ = max_t∈T_ {Δ(F/F_0_)_C_(t)}, where the maximum for each treatment T (max_t∈T_) is determined within the range of times defined by the addition of the corresponding treatment and that of the immediately subsequent one. Finally, both the normalized calcium response traces Δ(F/F_0_)_C_(t) and amplitudes Δ(F/F_0_)_C,T_ for the different treatments T were normalized, for each cell C, relative to the normalized calcium response amplitude upon ionomycin treatment for that cell, Δ(F/F_0_)_C,ionomycin_, prior to determining sample-averaged normalized traces and/or performing statistical analysis between different treatments/conditions. Sample-averaged traces (and their associated standard errors of the mean) were determined by averaging the ionomycin-normalized traces across all cells in each sample at every given time point. Because ionomycin normalization was performed on a per-cell basis, this necessarily results in the averaged traces not reaching a value of 1 at the end of the experiment (as this would require all cells in a sample to display their peak response to ionomycin at the exact same time point). Cells lacking a response to ionomycin were excluded from the analysis from the start, and are therefore not shown in the presented trace/quantification plots. We also excluded from the latter cells for which the fluorescence intensity response after addition of a given treatment did not drop back down to baseline levels before the addition of the immediately subsequent treatment. Of note, only about 1% of the total cells measured in our experiments were excluded due to either of these two criteria. Cells displaying a maximal treatment (aspartate/glutamate) response either higher than their respective ionomycin response or lower than baseline levels (and therefore having ionomycin-normalized maximal amplitudes above 1 or below 0 for that specific treatment) were on the other hand not excluded from the analysis, but rather interpreted as bona fide high/low treatment responders, respectively. Data from sequential additions of either aspartate or glutamate were further collected to ensure that sequential addition of two treatments would not result in NMDA receptor overactivation upon the first treatment and an ensuing ablation of the calcium response for the second, and are shown in Extended Data Figure 7p, Supplementary Movies 4,5.

### ^13^C-labeling from whole cell extracts and intracellular or cell receptor extracts

Spheroids were cultured in LLM supplemented with 700 μM of ^13^C_4_-aspartate or ^13^C_5_-glutamate (Cambridge Isotope Laboratories). For whole cell metabolite extracts, spheroids were harvested after 5 days and metabolites quenching and extraction were performed as previously described^28^. For intracellular cell fraction and cell receptor fraction metabolite extracts, spheroids were harvested after 5 days, washed 2x with quenching buffer and dissociated into single cells upon incubation with 200 μl of 0.25% trypsin EDTA (Thermo Fisher Scientific) at 37 °C for 15 minutes. Cell suspensions were subsequently centrifuged at 300 x g for 5 minutes to separate the supernatants (containing the cell receptor fractions) from the cell pellets (containing the intracellular fractions). The cell pellets were supplemented with 200 μl trypsin 0.25% and subsequently 800 μl of 100% MS-grade methanol containing the internal standards norvaline and glutarate (for a final concentration of 2.5 μg ml^-1^ at 1:1) were added to the cell pellet and the receptor fraction for metabolites extraction. Samples were centrifuged for 10 minutes at maximum speed at 4 °C and the supernatants containing the polar metabolites fraction were separated from the protein-containing pellets. The polar fractions were subsequently concentrated using a vacuum concentrator and the dried metabolite extracts were stored at -80 °C until mass spectrometry analysis. The protein pellet from the intracellular fraction was quantified by BCA and used for metabolite normalization of both the intracellular and receptor fractions.

To evaluate receptor fraction dissociation, cell pellets following 0.25% trypsin EDTA or StemPro Accutase (Thermo Fisher Scientific) digestion (performed 37 °C for 15 min), were incubated at 4 °C for 10 min with anti-mouse CD16/CD32 (Fc block, BD Biosciences, 553142). Samples were then incubated with anti-CD44 APC antibody (BioLegend, 103011, 1:500 dilution) and, to exclude dead cells, samples were stained with the Viability dye eFluor 450 (Thermo Fischer Scientific, 65-0863-14, 1:500 dilution) for 20 min at 4 °C. Samples were then analyzed using a BD FACSCanto II with FACSDiva software (BDBiosciences). The percentage of CD44-positive cells was used to assess the fraction of cell receptor present in the spheroids after dissociation using FlowJo software (as shown in Extended Data Figure 2l).

### Lung, liver and bone interstitial fluid collection

Lung interstitial fluid collection was adapted and performed as previously described^6,39,40^. For human samples, healthy lung tissue was harvested from patients undergoing lung surgery for emphysematous lung volume reduction. For mice samples, 6-8 weeks old BALB/C female mice were euthanized with 50 μl of a 60 mg ml^-1^ Dolethal (pentobarbital sodium) solution (Vetoquinol) and whole lungs or 200 mg liver were collected by surgical resection, washed with blood bank saline and dried from liquid excess. Bone interstitial fluid was collected by displacing femur and tibia and removing the patella. Tissues were then placed in a home-made filtered centrifugation tube supplemented with a 20 μm nylon mesh filter (Repligen). 1 to 10 μl of lung interstitial fluid was collected following centrifugation at 400 x g at 4 °C for 10 minutes and immediately stored on dry ice. The obtained interstitial fluid volume was used to determine the concentration of the polar metabolites measured by mass spectrometry.

### Brain cerebrospinal fluid collection

Mouse cerebrospinal fluid (CSF) was collected with a micropipette as previously described^41^. Briefly, a small subcutaneous incision was performed in the back of the head and cisterna magna was localized by visual inspection. Subsequently, the cisterna magna was punctured with a micropipette, avoiding brain parenchyma or blood vessels. Thanks to the capillary action, 4-7 μL of CSF flown into the micropipette and was stored on dry ice for mass spectrometry analysis.

### Lung and liver dissociation and flow cytometry analysis

4T1 and EMT6.5 cancer cells expressing the lentiviral vector pLKO.3 Thy1.1 (Addgene plasmid 1479) as a reporter protein were used for flow cytometry analysis. Cells expressing the surface protein Thy1.1 (CD90.1-positive cells) were sorted using FACSAria Fusion (BD Biosciences). For evaluating lung or liver metastatic burden, cells were injected in the m.f.p., i.v. or i.s. into 6-8 weeks old female BALB/C mice and after 21-24 days (m.f.p.), 13-17 days (i.v.) or 14 days (i.s.) mice were anesthetized with an i.p. injection of 10 μl g^-1^ containing ketamine (100 mg kg^-1^)-xylazine (10 mg kg^-1^). Lungs were perfused through the right ventricle, extracted and washed in blood bank saline. Livers were perfused through the left ventricle for full body perfusion, extracted and washed in blood bank saline. Tissues were then dried and minced with blades and subsequently incubated at 37 °C for 45 minutes with a solution of 0.3 mg ml^-1^ liberase (Roche) and DNase1 (1 μg ml^-1^) in 2 mL of RPMI supplemented with 5% fetal bovine serum. The dissociated lungs were quenched with 3% fetal bovine serum in PBS supplemented with 2mM EDTA, filtered through a 70 μm cell strainer and centrifuged for 5 minutes at 300 x g. The cell pellet was then washed, centrifuged for 5 minutes at 300 x g, incubated with Red Blood Cell Lysis buffer (Merck) and strained with a 40 μm cell strainer. The single-cell suspension was counted and 20x10^6^ cells ml^-1^ were incubated at 4 °C for 10 minutes with anti-mouse CD16/CD32 (Fc block, BD Biosciences, 553142). The samples were then stained at 4 °C for 20 minutes with fluorophore-conjugated antibodies against CD45 PerCP-Cy5.5 (BD Bioscience, 550994, 1:250 dilution), PDPN APC (BioLegend, 127409, 1:250 dilution) and CD90.1 Alexa Fluor 488 (BioLegend, 202505, 1:400 dilution). To exclude dead cells, samples were also stained with the Viability dye eFluor 450 (Thermo Fischer Scientific, 65-0863-14, 1:500 dilution). The samples were analyzed using a BD FACSCanto II with FACSDiva software (BD Biosciences). The percentage of metastasizing cells was measured by assessing the fraction of CD90.1-positive cells in the lung or liver using FlowJo software.

### Single-cell RNA sequencing

Female BALB/C mice were injected with TSF (*n* = 3) or control medium (*n* = 3) for three weeks, as described above, and subsequently either euthanized (pre-metastatic niche), or injected i.v. with 25x10^3^ CD90.1-expressing 4T1 cancer cells and then euthanized 11 days (metastatic seeding) or 16 days (metastatic colonization) after cancer-cell injection. Upon mice euthanasia, lungs were harvested and dissociated as described above. Cells were then pooled together from the 3 independent lung dissociations performed per group, resuspended in cell-culture medium at a density of 1x10^6^ cells ml^-1^, and kept on ice for immediate processing. Approximately 15 μl of each suspension were processed for single-cell library preparation, as previously described^6^, whereas the remainder of the single-cell suspension was processed in parallel for flow cytometry analysis, as described above. Barcoded single-cell cDNA libraries were generated using the Chromium Single Cell 5’ V1.1 Library Kit (10x Genomics), following the manufacturer’s guidelines, and aiming for a total of 10,000 cells per library. Single-cell libraries were sequenced on a NovaSeq 6000 System (Illumina), and the sequenced reads were then mapped to a customized version of the mm10 mouse genome (mm10 build GRCm38.p6, including an extra chromosome with the CD90.1 sequence used in the transduced lentiviral vector), using the Cell Ranger v5.0.1 software (10x Genomics). The resulting single-cell gene expression data were analyzed within the *R/Bioconductor* framework (www.r-project.org and www.bioconductor.org). Raw UMI count matrices for all samples were first imported using *Seurat* (v4.1.0)^42^ (www.satijalab.org/seurat), and immediately subject to ambient RNA correction using a customized version of the *SoupX* (v1.6.2) *R* pipeline (https://github.com/constantAmateur/SoupX)^43^. Specifically, two modifications were applied to the standard *SoupX* pipeline. First, the automatically determined (global) ambient contamination fractions for each of the samples were multiplicatively increased by a factor of 2, to account for the fact that, as reported in the literature, the automatic determination method tends to underestimate ambient contamination levels for single-cell RNA-sequencing data^44^. Second, rather than assuming a fixed contamination fraction for all cells, the latter was adapted for each cell based on its specific library size (total UMI counts), to achieve a uniform distribution of subtracted counts across all cells in each sample, in keeping with the nature of the ambient RNA contamination problem^44^. Ambient-corrected count matrices for the different samples were then merged and converted for further processing with *Monocle3-alpha* (v2.99.3)^45^ (www.github.com/cole-trapnell-lab/monocle-release/tree/monocle3_alpha). Low-quality cells were filtered out based on standard quality-control metrics, with thresholds chosen based on evaluating quality-control histograms for the merged data set. In particular, cells were filtered based on their mitochondrial RNA content (allowing for a maximum of 10%), library size (removing cells with total UMI counts below 800), and number of detected genes (removing cells expressing less than 200 genes). Genes not expressed in any of the cells remaining after quality-control filtering were ignored in all subsequent analyses. Size-factor and variance-stabilizing normalization (based on fitting to a negative binomial distribution) were then applied to the filtered data set, and highly variable genes (HVGs) were identified based on their departure from the average normalized dispersion *versus* expression trend observed among all genes. After excluding mitochondrial, ribosomal-protein, and cell cycle-associated genes, the top 1000 HVGs with size-factor normalized expression levels above 0.01 were selected. Principal component analysis (PCA) was then performed on the size factor-normalized and variance-stabilized count matrix restricted to these genes only, followed by 2D UMAP^46^ dimensional reduction based on the resulting top 50 principal components (with *correlation* distance metric, *number of neighbors* = 15, and *minimum distance* = 0.1, and without further PCA scaling). After that, cells were clustered in the UMAP plane by applying the *Louvain*^47^ graph-based algorithm at high resolution (resolution = 0.001, with *k*_*NN*_ = 7), in order to attain a fine-grained cluster structure (183 clusters). The resulting fine-grained clusters were then manually annotated to specific cell types, based on evaluating the expression profiles of several cell type-specific markers across the different fine-grained clusters. These cell-type annotations were further refined based on applying a second (analogous) step of dimensional reduction and (sub-)clustering analysis, separately to each of the preliminarily annotated cell-types. This in turn allowed us to more easily identify and filter out ambiguously annotated sub-clusters, most notably clusters originating from heterogeneous cell multiplets, characterized by simultaneously presenting markers associated to two or more cell types. Our multiplet identification was confirmed based on the distribution of doublet scores estimated across the different cells in each separate sample using the *R* package *scDblFinder* (v1.8.0)^48^ (https://bioconductor.org/packages/release/bioc/html/scDblFinder.html). Specifically for the case of cancer cells, the latter could be unambiguously identified upon the first clustering step based on CD90.1 expression, and no re-annotation or doublet removal were needed upon sub-clustering. Differential expression analysis, based on comparing cancer cells under TSF or control medium pre-treatment at 16 days of metastatic colonization only, was performed within the *Seurat* framework, using the function *FindMarkers* with default parameters other than preserving all genes in the output regardless of their determined fold-changes and/or their fraction of expressing cells across samples. The resulting (TSF *vs* control) log fold changes (log_2_ FC) and their associated p-values for each gene were then combined with the maximum fraction of cancer cells expressing that gene among the two conditions being compared (Xmax, where Xmax = 1 for genes expressed in 100% of cancer cells in either of the two conditions) to derive an adjusted ranking metric RM_adj_ = − log_2_ FC × log_10_(p-value) × X_max_, whereby highly positive/negative values of RM_adj_ indicate respectively genes with highly up/downregulated expression under TSF pre-treatment. Pre-ranked GSEA was then performed based on this adjusted ranking metric RM_adj_, and otherwise identically as described in the section Polysome profiling and sequencing analysis. Gene sets were ranked based on their normalized enrichment score (NES), with the top 15 gene sets with highest positive NES being shown in the manuscript. The NES plot corresponding to genes signature indicative of ECM remodeling was generated by extracting from the full gene-set collection all gene sets including either of the terms *COLLAGEN, MATRISOME, ECM*, or *EXTRACELLULAR_MATRIX*. The scRNA-seq raw data for the pre-metastatic niche were previously also used in^6^.

### Patient selection

For the collection of lung interstitial fluid from healthy subjects, all participants included in this study consented to participate. The study was approved by the local ethics committee (Medical Ethics Committee UZ/KU Leuven, protocol S57123). Sample collection was performed as described before^40,6^. Briefly, human lung healthy tissues was collected from patients undergoing lung surgery for emphysematous lung volume reduction (Supplementary Table 4) and lung interstitial fluid was collected as described above.

### UPTIDER patients

Snap-frozen, paraffin embedded and fresh tissue samples from lung metastases and healthy tissues were obtained through the ethically approved UPTIDER program (UZ/KU Leuven Program for *Postmortem* Tissue Donation to Enhance Research, NCT04531696 S64410). Patients with metastatic breast cancer who consented to participate in UPTIDER were included in our study. Lung interstitial fluid was collected from fresh tissue samples, proteins from metastatic and non metastatic lungs were extracted from snap-frozen tissues and IHC was performed from paraffin embedded tissues, as described above. Clinicopathological information for every patient in the UPTIDER dataset is shown in Supplementary Table 5.

### H&E, Picrosirius Red staining and Birefringence analysis

Haematoxylin and eosin (H&E) staining of lung was performed to identify metastatic lesions, while Picrosirius red staining was used to image fibrillar collagen in lung metastatic lesions. Female BALB/C mice were euthanized with 50 μl of a 60 mg ml^-1^ Dolethal (pentobarbital sodium) solution (Vetoquinol) and lungs were infused through the trachea with 10% neutral-buffered formalin. Tissues were then incubated overnight in 10% neutral-buffered formalin and transferred into 70% EtOH. Tissues were further processed and embedded into paraffin using Histokinette STP 120-2 (Fisher scientific). Afterwards, lung samples were deparaffinized, rehydrated and sliced sections of 7 μm were stained with H&E or with 0.1% picrosirius red (Polysciences)^9^. H&E stainings were performed with Leica Autostainer XL (ST5010) with manufacture’s recommended H&E protocol. H&E images were scanned on a Zeiss Axio Scan.ZI (20x) with a ZEN software (v3.4) and the analysis was performed using the ZEN Blue software (Zeiss). Lung metastatic burden was quantified by analyzing the total metastasis area. For picrosirius red staining, images were acquired with AxioScan 7 equipped with polarized light source (20x) with a ZEN software (v.3.4). For human samples, picrosirius red tissue were counterstained with Weigert’s hematoxylin to identify metastatic regions, as described in^49^. For fibrillar collagen analysis, individual metastases were identified and isolated in the birefringence images; area that included blood vessels or lungs rim were excluded from our analysis due to their endogenous collagen enrichment. Quantitative measurements of fibrillar collagen I signal (red), collagen III (green) and collagen I/III (yellow) were carried out in ImageJ software using an in-house script as previously published^50^. For each image, Hue-Saturation-Balance (HSB) thresholding was applied, where 2≥H≤27 | 0≥S≤255 | 140≥B≤255 was used for red-orange (high birefringent) fibers, 28≥H≤47 | 0≥S≤255 | 140≥B≤255 for yellow (medium birefringent) fibers, and 48≥H≤140 | 0≥S≤255 | 140≥B≤255 for green (low birefringent) fibers. The relative area (as a percentage of total tissue area) was then calculated.

### Multiplex immunohistochemistry staining of human and mice lungs

For multiplex immunohistochemistry the OPAL 6 plex kit (Akoya) was used. Each primary antibody dilution was optimized in a single staining and subsequently, optimal position of antibody was determined in a multiplex staining. In details, 7 μm thick slices of tissue previously embedded as described in H&E section were cut and mounted on BOND Plus Slides (Leica). Slides were dried for 16h at 37 °C and then stored at RT until experiment. All the staining steps were performed with the BOND RX Fully Automated Research Stainer 21.2821 (Leica). On day of experiment tissue was deparaffinized with DEWAX solution (Leica) followed by antigen retrieval with AR6 (Leica). Tissues were then blocked with blocking buffer (10% goat serum, Invitrogen, 0.5% BSA in TBS Buffer) for 30 min. Primary antibodies were diluted in TNB (Tris-NaCl Blocking buffer): 100 mM of Tris-HCl pH 7.5, 150 mM NaCl, 0.5% w/v of blocking reagent (Akoya, FP1020). Subsequently, slides were incubated for 1h at RT with the corresponding antibody, rinsed twice with BOND wash buffer (Leica) followed by three washes of 5 min each with BOND wash buffer. Corresponding secondary HRP (Dako rabbit or Opal kit for mouse HRP) were incubated for 10 min followed by two rinses and two washes of 5 min with BOND wash solution. After the washes, the OPAL dye was incubated for 15 min. The slides were then washed again and followed by either DAPI (Spectral DAPI Akoya) or in case of multiplex staining the next round of staining starting by antigen retrieval for 10 min at 100 °C, blocking, primary antibody, washes, HRP antibody, washes, OPAL dye, washes, DAPI. Afterwards, the staining slides were removed from the autostainer and mounted with antifade diamond mounting media (Invitrogen) and imaged using AKOYA Phenoimager. Spectral unmixing was performed online with standard library provided by AKOYA Version 2.0 onwards and unmixed images were analysed using Qupath Version 0.5 onwards. The following primary antibodies were used: hypusine (EMD Millipore, ABS1064, 1:50 dilution for mouse tissues, 1:75 dilution for human tissues), phosphorylated SMAD3 (S423+S425) (Abcam, EP823Y, 1:100 dilution), phosphorylated CREB (Ser133) (Cell Signaling Technologies, 9198S, 1:200), DOHH (Sigma-Aldrich, HPA041953, 1:300 dilution), Grin2d (Novus Biological, NBP2-94573, 1:300 dilution), PanCK (Dako, M3515, 1:200 dilution), EpCAM (Abcam, Ab71916, 1:1000 dilution), Collagen I (Cell Signaling Technologies, E8F4L XP® 1:300), Collagen VI (Abcam, Ab182744, 1:1,000 dilution) and for membrane stain detection ATP1A1 (Proteintech, 14418-1-ap 1:200 dilution) and Wheat Germ Agglutinin, HRP Conjugate (Biotium, Inc, 29073, 1μg mL^-1^). For image analysis, metastases were detected using EpCAM for mouse tissue and PanCK for human tissue, followed by the analysis of the stainings for hypusine, DOHH, phosphorylated SMAD3, COL6A1, Grin2d, phosphorylated CREB and COL1A1.

For mouse tissue, quantification for hypusine, Dohh, phosphorylated SMAD3, Grin2d and phosphorylated CREB was performed by determining the mean fluorescence intensity per unit area across 5 independent regions (all EpCAM positive), or as percentage of positive cells (only for Dohh and hypusine) per region in 5 different metastases. For Col6a1 and Col1a1, which can be secreted and accumulated in surrounding EpCAM positive areas, quantification was performed by determining the mean fluorescence intensity per unit area across 5 independent regions (including EpCAM positive area and closely surrounding area). If blood vessel and tissue borders were present in the selected area, Col1a1 and Col6a1 signals resulting from those endogenously high-collagen regions were excluded from the analysis. The following multiplex combinations were done: Grin2d, membrane marker, phosphorylated CREB, EpCAM, Col1a1. Dohh, hypusine, phosphorylated SMAD3, Col6a1 and EpCAM.

For human tissue, quantification for Hypusine, DOHH, phosphorylated SMAD3, GRIN2D and phosphorylated CREB was performed by determining the fluorescence intensity per cell (Hypusine, DOHH and GRIN2D) or per nucleus (phosphorylated SMAD3 and phosphorylated CREB) across 5 independent regions for each metastatic (PanCK positive) or adjacent tissue. Statistical analysis was performed based on fluorescence intensity values per single cell. For COL6A1 and COL1A1, which can be secreted and accumulated in surrounding PanCK positive areas, quantification was performed by determining the mean fluorescence intensity per unit area across 5 independent regions for each metastatic (PanCK positive) or adjacent tissue. Statistical analysis was performed based on fluorescence intensity values per unit area. If blood vessel and tissue borders were present in the selected area, COL1A1 and COL6A1 signals resulting from those endogenously high-collagen regions were excluded from the analysis. For representative images visualization, fluorescent tissue background was subtracted using the intensity values of the AF (autofluorescent channel) obtained during tissue scanning in the AKOYA Phenoimager. Representative images from patients quantified in Extended Data Figure 5c and corresponding H&E stainings of the whole lung sections are shown in Figure 5e and Extended Data Figure 6.

### Mass spectrometry

Polar metabolites were analyzed by gas chromatography (GC) or liquid chromatography (LC) mass spectrometry. For polar metabolites measurements analyzed by GC, dried metabolites samples were derivatized as previously described^9^. Isotopologue distributions and metabolite concentrations were measured using a 7890A GC system (Agilent Technologies) combined with a 5975C Inert MS System (Agilent Technologies) or a 8860 GC system combined with a 5977C Inert MS System (Agilent Technologies). The inlet temperature was set at 270 °C and 1 μl of sample was injected into a DB35MS column with a split ratio of 1 or 3 to 1 or 9 to 1. The carrier gas flow of helium was fixed at 1 ml min^-1^. After the injection, the GC oven was kept at 100 °C for 1 min, increased up to 105 °C with a gradient of 2.5 °C min^-1^ for 2 min, then ramped to 240 °C with a gradient of 3.5°C min^-1^, and after that ramped up to 320 °C with a gradient of 22 °C min^-1^, which was followed by 4 min at 320 °C for 2 min. Mass spectrometry was performed at 70 eV and a mass range of 150-650 atomic mass units was measured. Data was collected using MSD Chemstation Data Analysis software. Metabolites abundances and isotopologue distributions were extracted from raw chromatograms, corrected for naturally occurring isotopes and normalized to the internal standard and protein content (for cell extracts) or volume (for fluid extracts) with a MATLAB Script (MA, USA). A standard curve for each metabolite was used to calculate polar metabolite concentrations. The various standard curve dilutions were extracted and run in parallel with the sample. For polar metabolite measurements analyzed by LC–MS, a 1290 Infinity II with a thermal autosampler set at 4 °C, coupled to a Q-TOF 6546 mass spectrometer (Agilent Technologies) was used. Dried metabolites sample were resuspended in 80% methanol:water and a volume of 1-10 μl of sample was injected into an Agilent InfinityLab Poroshell 120 HILIC-Z column, 2.1 mm × 150 mm, 2.7 μm, PEEK-lined. For positive mode analysis, the separation of metabolites was achieved at 25°C with a flow rate of 0.25 ml min^-1^. A gradient was applied for 23 min (solvent A: 10 mM ammonium formate in water with 0.1% formic acid; solvent B: 10 mM ammonium formate in water/acetonitrile 10:90 (v:v) with 0.1% formic acid) to separate the targeted metabolites (0 min: 98%B, 3 min: 98%B, 11 min: 70%B, 12 min: 60%B, 16 min: 5%B, 18 min: 5%B, 19 min: 98%B; 23 min: 98%B). The MS was operated in positive full scan mode (m/z range: 50-1200) using a sheath gas temperature of 225 °C (10L min^-1^) and a gas temperature at 225 °C (6L min^-1^). The nebulizer was set at 40 psi, the fragmentor at 125V and the capillary at 3000 V. For negative mode analysis, the separation of metabolites was achieved at 50 °C with a flow rate of 0.25 ml min^-1^. A gradient was applied for 32 min (solvent A: 10 mM ammonium acetate in water with 2.5 μM InfinityLab Deactivator Additive, pH = 9 – solvent B: 10 mM ammonium acetate in water/acetonitrile 15:85 (v:v) with 2.5 μM InfinityLab Deactivator Additive, pH = 9 or 15 mM ammonium acetate in water/acetonitrile 15:85 (v:v) with 2.5 μM InfinityLab Deactivator Additive, pH = 9) to separate the targeted metabolites (0 min: 96%B, 2 min: 96%B, 5.5 min: 88%B, 8.5 min: 88%B, 9 min: 86%B, 14 min: 86%B, 19 min: 82%B; 25 min: 65%B, 27 min: 65%B, 28 min: 96%B; 32 min: 96%B). The MS operated in negative full scan mode (m/z range: 50-1200) using a sheath gas temperature of 350 °C (12L min^-1^) and a gas temperature at 225 °C (13L min^-1^). The nebulizer was set at 35 psi, the fragmentor at 125V and the capillary at 3500 V. Data was collected using the MassHunter Workstation LC/MS Data Acquisition v.10.1 Build 10.1.48 (Agilent Technologies) and was analyzed using the Agilent MassHunter Workstation Profinder v.10.0.2.Build 10.0.2.162. Data were normalized to internal standard and protein content (for cell extracts).

### *In silico* patient gene expression data analysis

To compare gene expression profiles in patient-derived breast cancer metastases at different organ sites, we downloaded the microarray-based data set GSE14018^51^ (https://www.ncbi.nlm.nih.gov/geo/query/acc.cgi?acc=GSE14018) from the Gene Expression Omnibus (GEO) using the *R* package *GEOquery* (v2.62.2). Data for probes with undefined gene symbols or with ambiguous gene assignments (i.e. annotated to multiple different genes) were filtered out before further analysis. Among the remaining probes, those presenting duplicated gene symbols were further collapsed into single probes, by preserving only those probes with the highest overall expression levels for each duplicated gene symbol. A total of 12502 probes were considered in the final analysis after these filters. Differential expression analysis was performed based on the provided RMA-normalized expression levels for these probes, using the *R* package *limma*^52^ (v3.50.1) (https://bioinf.wehi.edu.au/limma), and comparing expression levels in 16 Lung samples *vs* 20 samples from all other metastatic sites available in the data set (5 Liver, 8 Bone, and 7 Brain samples, all considered together). The resulting (Lung *vs* Other) log fold changes (log_2_ FC) and their associated p-values were then combined to derive a ranking metric RM = − log_2_ FC × log_10_(p-value), whereby highly positive/negative values of RM indicate respectively genes with highly up/downregulated expression in Lung *vs* all other metastatic sites. Pre-ranked GSEA was then performed analogously as described in the section Polysome profiling and sequencing analysis, only based on this ranking metric RM and considering human gene sets.

### Statistical analysis

Statistical data analysis was performed using GraphPad Prism v.9 and v.10 (GraphPad Software) or within the *R/Bioconductor* framework on *n* ≥ 3 biologically independent replicates. Data are presented as mean ± s.d, mean ± s.e.m, or median ± 95% confidence interval, as indicated in the figure legends. Details on statistical tests and post-hoc tests are indicated in the legends, non-significant comparisons are not shown. *P* values <10^-4^ are indicated as *P* < 0.0001. Mathematical outliers were determined using the Grubbs’ or ROUT method of regression (GraphPad) with alpha = 0.05 or coefficient *Q* = 1%. Independent experiments were pooled and analyzed together whenever possible as described in the figure legends. For *in vivo* experiments, mice were randomized before control/TSF injection and PBS/aspartate injection or injection with the different cell lines. Mice were assigned to unique number before data collection for blinded analysis. For *in vitro* studies, samples were randomized when possible before data acquisition.

## Resource Availability

### Lead Contact

Further requests for resources should be directed to the lead contact, Sarah-Maria Fendt (sarah-maria.fendt@kuleuven.be).

## Materials Availability

This study did not generate new unique reagents, except of genetically manipulated cell lines based on commercially available constructs. Reagents generated in this study will be made available on request through the lead author or the collaboration partner that generated the resource, but we may require a payment and/or a completed Materials Transfer Agreement if there is potential for commercial application.

## Extended Data

## Data Availability

Mouse single-cell RNA-sequencing data and polysome/total RNA sequencing data generated as part of this study have been deposited in the Gene Expression Omnibus (GEO) under accession code GSE236087. The publicly available microarray-based patient-metastasis data set GSE1401882 can be downloaded from the Gene Expression Omnibus (GEO) https://www.ncbi.nlm.nih.gov/geo/query/acc.cgi?acc=GSE14018. All other data supporting the findings of this study are available within the Article and the Supplementary Information, and from the corresponding author on reasonable request. No original softwares and/or algorithms were developed in the present study; however, code used for data analysis can be provided upon request. Any additional information required to reanalyze the data reported in this paper is available from the corresponding author upon request.
